# Process mapping in clinical healthcare settings in low-income and lower-middle-income countries: a scoping review

**DOI:** 10.3389/phrs.2026.1609105

**Published:** 2026-10-14

**Authors:** Judith Boegli, Karin Gross, Fenella Beynon, Leah F. Bohle

**Affiliations:** 1 Swiss TPH, Allschwil, Switzerland; 2 University of Basel, Basel, Switzerland

**Keywords:** healthcare improvement, LMICs, process mapping, quality of care, scoping review, patient journey

## Abstract

**Objectives:**

To examine the use of process mapping (PM) in clinical healthcare settings in low- and lower-middle-income countries (LLMICs).

**Methods:**

A scoping review was conducted. Full-text empirical studies published from 2014 to 2024 were identified from MEDLINE, Embase, Web of Science, and CINAHL databases.

**Results:**

Twenty-six articles were included. Deductive and inductive content analysis was performed. Applications have included process- and quality-improvement initiatives (n = 12), health system process analysis and assessment (n = 7), health systems tools design, implementation and evaluation (n = 4), and costing (n = 3). Observations, workshops and interviews were applied, with 65% using a single method (n = 17). PM was predominantly researcher-led (n = 17), with local healthcare workers participating in the majority of studies (n = 21); none involved patients. Gaps in reporting and documentation of PM activities were identified (n = 16).

**Conclusion:**

PM demonstrated significant potential as a versatile and adaptable method for improving clinical processes. Future PM studies should adhere to reporting guidelines, promote locally led PM activities and incorporate diverse perspectives, particularly those of patients.

## Introduction

In the pursuit of universal health coverage (UHC), delivering and improving high-quality services is of great importance and should remain on the international health agenda [1]. Studies have shown that, especially in low- and lower-middle income countries (LLMICs), poor quality healthcare services have a significant impact on mortality and morbidity rates [1–3].

Process mapping (PM), with its systems thinking approach, is a quality improvement methodology that can address the challenge of poor-quality healthcare services in LLMICs. Originating in the manufacturing industry in 1930 [4, 5], it is now extensively applied in healthcare to assess, analyse and improve processes with a focus on enhancing quality of care [6]. While this methodology is commonly used in high-income countries [7–11], it has not yet been widely applied in LLMICs [12–14], where healthcare quality improvements are urgently needed [2]. As it can be applied with limited infrastructure and personnel, PM is a valuable tool to support efficient and effective changes in LLMICs [13–15].

In this review, PM is defined as “an entire approach that leads to a holistic understanding of the process under review” [6, 16]. It provides a realistic understanding of a process, allows evaluation of variation from standard practices and helps to identify opportunities for improvement [8, 17]. PM creates a visual representation of a process under review, showing one step after another [18]. While it is also widely used to improve surveillance data, such as civil registration and statistics [19, 20], this review focuses only on its application in clinical healthcare settings.

Being a versatile methodology, PM can be applied to various types of processes in clinical healthcare settings [8]. It can map a patient flow in a specific clinical setting, for example in an emergency department, from arrival through to waiting and examination rooms, until discharge or referral [21]. Such process maps allow analysis and understanding of different steps in a pathway, workflow or patient journey and help to identify potential inefficiencies and opportunities for improvement in the process of care under review [8].

A systematic review by Antonacci et al. in 2021 [7] proposed a comprehensive PM-framework comprising the following phases: 1) “Preparation”, 2) “Data and information gathering”, 3) “Process map generation”, 4) “Process map analysis” and 5) “Take it forward” [7]. During these phases, data collection can involve various qualitative research methods, such as interviews, observations, and workshops.

These methods have the potential to be participatory and adaptable for local contexts, which may be relevant to LLMICs. However, to date no review has systematically examined if, when and how these methods are used in PM activities in such settings.

There has been growing emphasis on involving local healthcare workers and stakeholders in healthcare improvement initiatives to ensure their effectiveness, sustainability, and acceptability while addressing barriers with tailored context-specific solutions [22, 23]. Due to its bottom-up approach, PM is well suited for local participation [8, 24, 25]. Yet there is a lack of evidence on the role and involvement of local healthcare workers in PM activities.

PM also has potential for advancing implementation science. The final phase of PM proposed by Antonacci et al. [7] “Take it forward” focuses on implementing proposed actions and changes. This makes PM an invaluable tool for bridging the gap between research and tangible local-level action. The importance of implementation science has become increasingly evident, especially in LLMICs [26, 27].

Despite growing interest in PM as a tool for improving healthcare delivery, there remains little synthesis of how it is being applied in LLMICs, particularly with regard to implementation approaches, stakeholder involvement, and follow-through into system change.

This scoping review aims to provide a comprehensive overview of the use and implementation of PM in clinical healthcare settings in LLMICs.

It entails an analysis of the key characteristics of healthcare contexts, clinical specialities, the overarching objectives and types of processes reported in the existing PM literature. Moreover, this review explores the use of methods in PM activities, the extent of local stakeholder involvement and the level of implementation.

## Methods

This scoping review followed the Joanna Briggs Insitute (JBI) Manual for Evidence Synthesis for scoping reviews [28] and adhered to the Preferred Reporting Items for Systematic Reviews and Meta-Analyses (PRISMA-ScR) framework [29]. No formal protocol was registered.

### Search strategy

A comprehensive search strategy based on the terms “process mapping” (PM) and “low- and lower-middle-income countries” (LLMICs) targeted published articles in four databases: “Medline”, “Embase”, “Web of Science Core Collection” (WoS), and “Cumulative Index to Nursing and Allied Health Literature” (CINAHL), see Supplementary Material 1.

To account for variations in terminology, comparable terms for *PM* were incorporated into the search strategy.

The Cochrane Effective Practice and Organisation of Care (EPOC) filters for low- and middle-income countries (LMICs) served as a starting template [30]. However, since these filters were not up to date, they were manually adapted to the current list of LLMICs as defined by the World Bank [31].

For the interdisciplinary “WoS” database which also includes non-health literature, a third search term “clinical healthcare settings” was defined. Comparable terms such as “health service” and “health facilities” were included.

See detailed search strategy in Supplementary Material 1.

The eligibility criteria followed the Participant, Concept, Context (PCC) framework [29, 31], see Table 1. As this review focused on methodology, the criterion “type of participants” was not included. English language, peer-reviewed primary research articles, published from 01.01.2014 to 31.05.2024 were included to capture recent evidence on PM use. The final search was on 26.06.2024.

**TABLE 1:** Inclusion/Exclusion criteria (Low-income and lower-middle- income countries, 2014–2024).

Inclusion criteria	
Concept	Description of PM/PM activities
	Description of procedures and steps in a PM/PM activity
Context I	Conducted in a low-income and lower-middle-income countries as defined by the World Bank [31]
Context II	Performed in clinical healthcare settings (human only)
Language	Full text published in English
Time	Published between 01.01.2014 - 31.05.2024
Source	Full-text empirical studies, conducted with primary research data collection
Exclusion criteria	
All studies which did not meet the aforementioned inclusion criteria were excluded. Two further exclusion criteria were applied	
Context I	Studies where the location was not identified following a full-text screening
Source	Studies which were not full empirical studies (incl. Methodological studies, conference proceedings, posters and grey literature)

### Screening and selection

All articles (n = 2621) were imported into Rayyan web application [32], duplicates were removed. Title and abstract were screened against the eligibility criteria. Full-text screening was done independently and blinded by two authors, who assessed the eligibility of the remaining studies (See Supplementary Material 2). Papers were included if they fully described the procedures/steps of their PM activities and the process of how they conducted their process maps [12, 14, 17, 21, 33–54]. Varying definitions of the term “*PM*” in the literature led to some conflicting evaluations. These conflicts were resolved in a meeting between JB, LFB and FB, during which Antonacci’s definition of PM [7] was reaffirmed, along with the inclusion criteria.

### Data extraction and analysis

Data were extracted by JB using an extraction sheet in Microsoft Excel, which was pilot-tested with five included studies (Supplementary Material 3). Based on this pilot, the sheet was revised to better capture the range of methodologies used by the included studies to conduct PM, by introducing separate sections for each data collection method (workshops, observations, interviews), with distinct variables within each section. Data extraction and charting were performed by JB with uncertainties discussed with the co-authors.

Data analysis aimed to categorise study characteristics, such as geographic location, health system level, and clinical settings (e.g. inpatient or outpatient). To categorise the clinical specialities, the WHO standard classification of health categories [55] and a previous scoping review that similarly categorised disease and treatment specialities [9] were used as references with adaptions for the purpose of this review.

See Supplementary Material 4 for further details on this adaptation process and the charting and coding scheme.

An inductive qualitative content analysis was initially conducted to examine how PM was conducted.

To identify the different types of processes subject to PM, an inductive category development was subsequently conducted through open coding and grouping similar process descriptions, guided by the coding question “What type of process was mapped?”.

To analyse the studies regarding their overarching objectives and PM phases, deductive structures [56], guided by Antonacci’s comprehensive framework and its phases [7], were combined with an inductive approach to allow new categories and codes to emerge.

Studies were assigned to one of four overarching objective categories: health systems process analysis and assessment (inductive), process- and quality-improvement initiatives (deductive), health systems tools design, implementation and evaluation (deductive), and costing (deductive).

Within the PM phases, emphasis was given to the phase “Data and information gathering” in which the use of the methods (workshops, observations and interviews) was analysed to capture methodological specifications. Additional elements, such as the extent of participation and involvement of local healthcare providers were included to evaluate how PM activities were conducted in clinical healthcare settings. Extracted data were summarised, descriptively and narratively, structured around the predefined coding framework.

Each category was defined according to explicit decision rules; the full coding scheme, decision rules and a coding tree are provided in Supplementary Material 4. Consistent with current guidance for scoping review methodology [29, 56], no formal critical appraisal or risk-of-bias assessment of the included studies was undertaken; instead, this review focused on mapping the breadth of the available evidence. Reporting and documentation were assessed in terms of completeness against predefined extraction variables, rather than methodological quality or risk of bias. In Supplementary Material 5, a completed PRISMA-ScR checklist [29] on how this review was reported can be found.

## Results

A total of 2621 articles were identified. After duplicates were removed, 1570 articles remained for title and abstract screening, resulting in 1494 articles being excluded (Figure 1). Full-text screening assessed eligibility of the remaining 76 studies (See Supplementary Material 2). Of these, 26 papers fulfilled the inclusion criteria [12, 14, 17, 21, 33–54].

**FIGURE 1:**
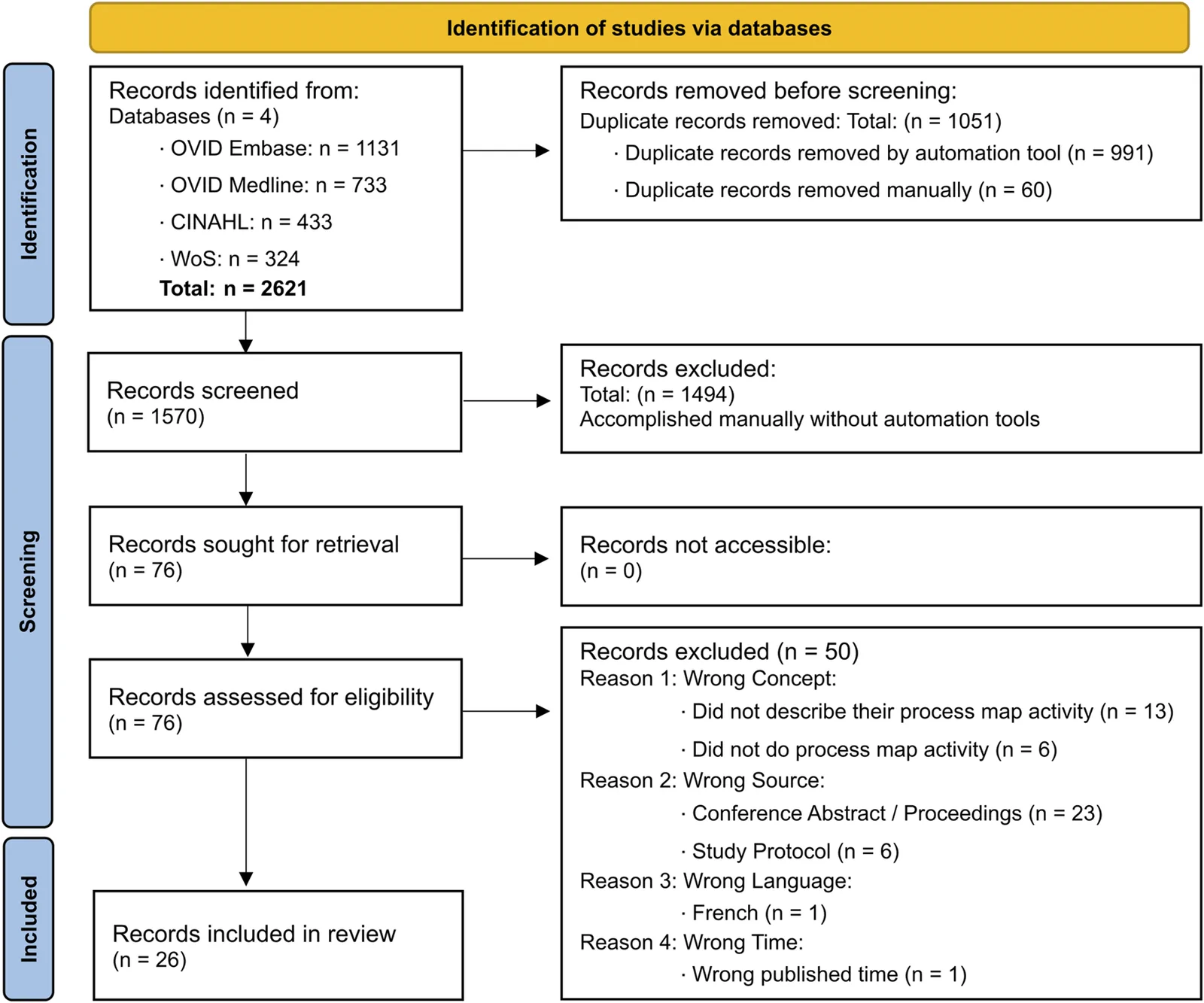
PRISMA flow diagram 2020 of publications on process mapping in LLMICs (low-income and lower-middle-income countries, 2014-2024). Adapted from the PRISMA 2020 flow diagram template [57], licensed under CC BY 4.0.

### Study characteristics

The 26 studies included in this review exhibited considerable variation in their characteristics (Figure 2).

**FIGURE 2:**
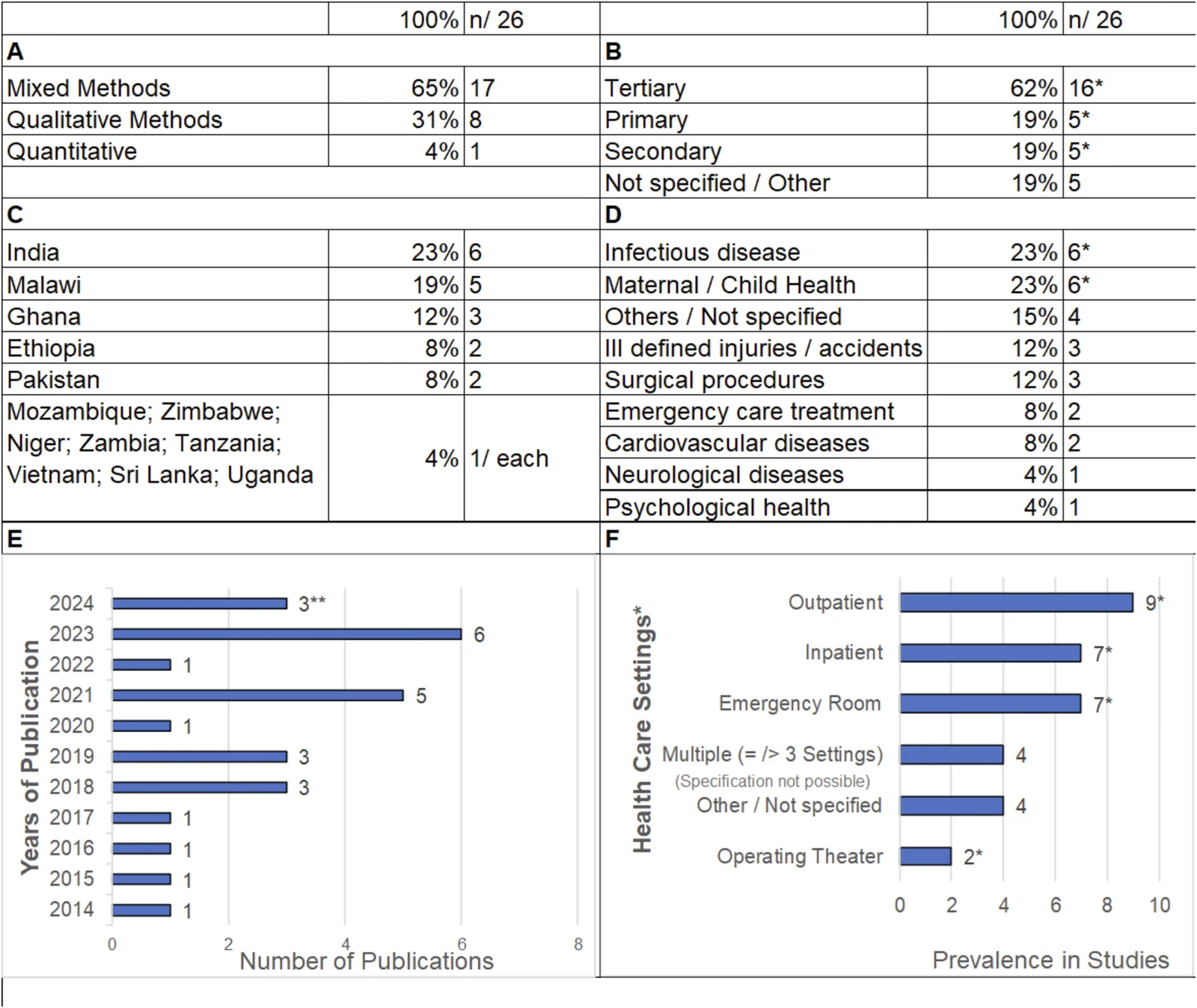
Characteristics of process mapping studies I Diagram created with Microsoft Excel [58]. *Some studies were classified into multiple categories; percentage indicated proportion of total 26 studies. I **Publication included until May 2024. **(A)** Type of research method. **(B)** Health system level. **(C)** Country studies were conducted in. **(D)** Panel D presents clinical topics, featuring infectious disease and maternal/child health being the most prevalent. **(E)** Years of publication. **(F)** Healthcare settings (Low-income and lower-middle- income countries, 2014–2024).

Only 13 of 81 LLMICs (16%) were represented: India had the highest number (n = 6; 23%), followed by Malawi (n = 5; 19%) and Ghana (n = 3; 12%), as shown in Figure 2C. The majority of the studies were conducted at the tertiary level (n = 16; 62%) [12, 14, 33, 35, 36, 38, 39, 41–44, 46, 47, 51, 52, 54]. Infectious disease (n = 6; 23%) [12, 37, 40, 45, 46, 50] and maternal and child health (n = 6; 23%) [40, 43, 47, 48, 50, 51] were the most common focus. The number of publications on PM has increased over the past 5 years, with a peak of six studies published in 2023 (n = 6; 23%).

### Overarching objectives of conducting PM

Four categories of overarching objectives were identified (Figure 3A), with the most common being “Process- and quality-improvement initiatives”, identified in nearly half of the studies (n = 12; 46%) [12, 14, 17, 21, 33, 39, 41, 48, 49, 51–53]. This category comprised studies whereby the primary aim was to improve processes within a process- and/or quality improvement initiative. Of these, the studies were classified according to their methodological approaches, including “Plan-Do-Study-Act Cycle” (PDSA) (n = 4; 15%), “Lean Management” (LEAN) (n = 1; 4%), “A3 Problem Solving” (n = 1; 4%) and “Failure Mode and Effects Analysis” (FMEA) (n = 1; 4%). One study aimed to solely assess the process under review (n = 1; 4%), while four other studies did not specify (n = 4; 15%).

**FIGURE 3:**
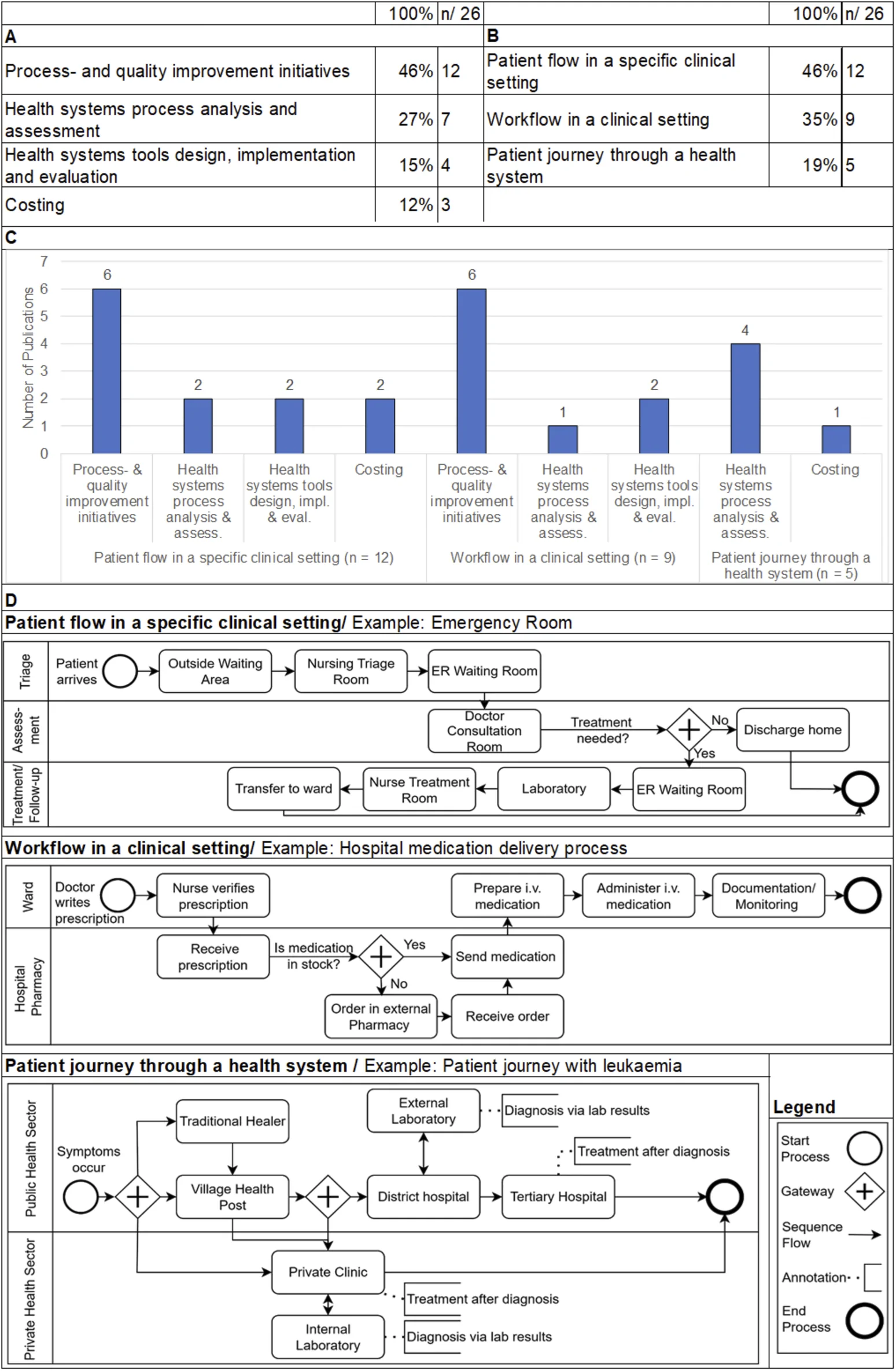
Different types of processes I Diagram created with Microsoft Excel [58] I Illustration created with diagrams.net [59]. **(A)** Overarching objectives. **(B)** Type of processes being observed. **(C)** Synthesis overarching objectives of studies with types of processes. **(D)** Different types of processes. (Low-income and lower-middle- income countries, 2014–2024).

Studies with the objective of “Health systems process analysis and assessment” accounted for 27% (n = 7) of the included studies and primarily aimed to identify, understand and map processes or procedures in a certain healthcare system [34, 42, 43, 47, 50, 53, 54].

“Health systems tools design, implementation and evaluation”, which used PM to support the design, implementation and/or evaluation of tools (digital and non-digital) in a specific health system, was represented in 15% (n = 4) [35, 36, 46, 60]; two of these (8%) implementing digital tools [35, 46].

The smallest category, 12% (n = 3) [37, 44, 45] aimed to map “Costing” with the widely recognised approach “Time-Driven Activity-Based Costing (TDABC)”, which provides a comprehensive estimation of patient-related costs, that encompass personnel, logistical and infrastructural expenses [61].

### Types of processes being observed

Three different types of processes observed during PM were found (Figure 3 (B; D)). “Patient flow in a specific clinical setting” was observed in 12 studies (46%) [12, 14, 21, 401, 42, 44–46, 48, 50, 52, 60]. This involved “shadowing” and mapping the different processes and steps of a patient’s flow in a clinical setting to create a process map. The clinical setting was in 23% the emergency room (n = 6) [12, 14, 21, 37, 41, 42], followed by outpatient settings (n = 4; 15%) [37, 48, 50, 52].

A “workflow in a clinical setting” was observed in 35% (n = 9), aiming to map a workflow or standard procedure [17, 33, 35, 36, 38, 39, 43, 49, 51], including hospital payment processes [38], infection prevention workflows [39], perioperative standard care [17], and observing and improving work processes in a home care delivery team [49]. Most were conducted in inpatient settings [36, 39, 43, 51, 53], though two spanned both inpatient and outpatient settings [35, 43].

Lastly, 19% (n = 5) performed PM to analyse a “patient journey through a health system” in relation to a specific symptom/disease [34, 37, 47, 53, 54].

One study examined patients‘ journeys with breast disease navigating through different hospitals in the Niger health system [54], while another mapped the process of care and cost-related factors for the treatment of tuberculosis in the Zimbabwean healthcare system [37]. One study focused on the processes in the emergency obstetric referral system in Ghana [47]. Two studies (8%) (that were part of the same project) observed the journey of individuals who sustained injuries in the Malawian health system [34, 53]. As Figure 3C illustrates, four (15%) of five studies had the overarching objective of “Health systems process analysis and assessment”.

### PM phase analysis

Variable reporting of PM activities was found with respect to the phases outlined in Antonacci’s framework [7].

### Phase 1: preparation

35% of studies (n = 9) reported on preparation activities in the local setting, prior to initiating PM activities. These included providing detailed information about PM as a methodology, an overview of the PM activities, and/or an introduction of the study teams to the local stakeholders [12, 14, 33, 36, 39, 41, 42, 45, 53].

### Phase 2: data and information gathering, including local stakeholder involvement

Among the reviewed publications, 17 studies used one method for collecting PM data (65%) [12, 17, 21, 34, 35, 37, 40–45, 49, 50, 52–54], while six studies combined two methods (n = 6; 23%) [35–38, 41, 48], see Figure 4A. The most prevalent PM methods included workshops (n = 6; 23%), observations (n = 15; 58%), and interviews (n = 8; 31%) either in combination or stand-alone. Three studies (12%) did not provide detailed information on how they gathered data for their process map [34, 51, 60]. Notable variation emerged when analysing who led the PM exercise, and the degree of local healthcare involvement (see Figure 4B).

**FIGURE 4:**
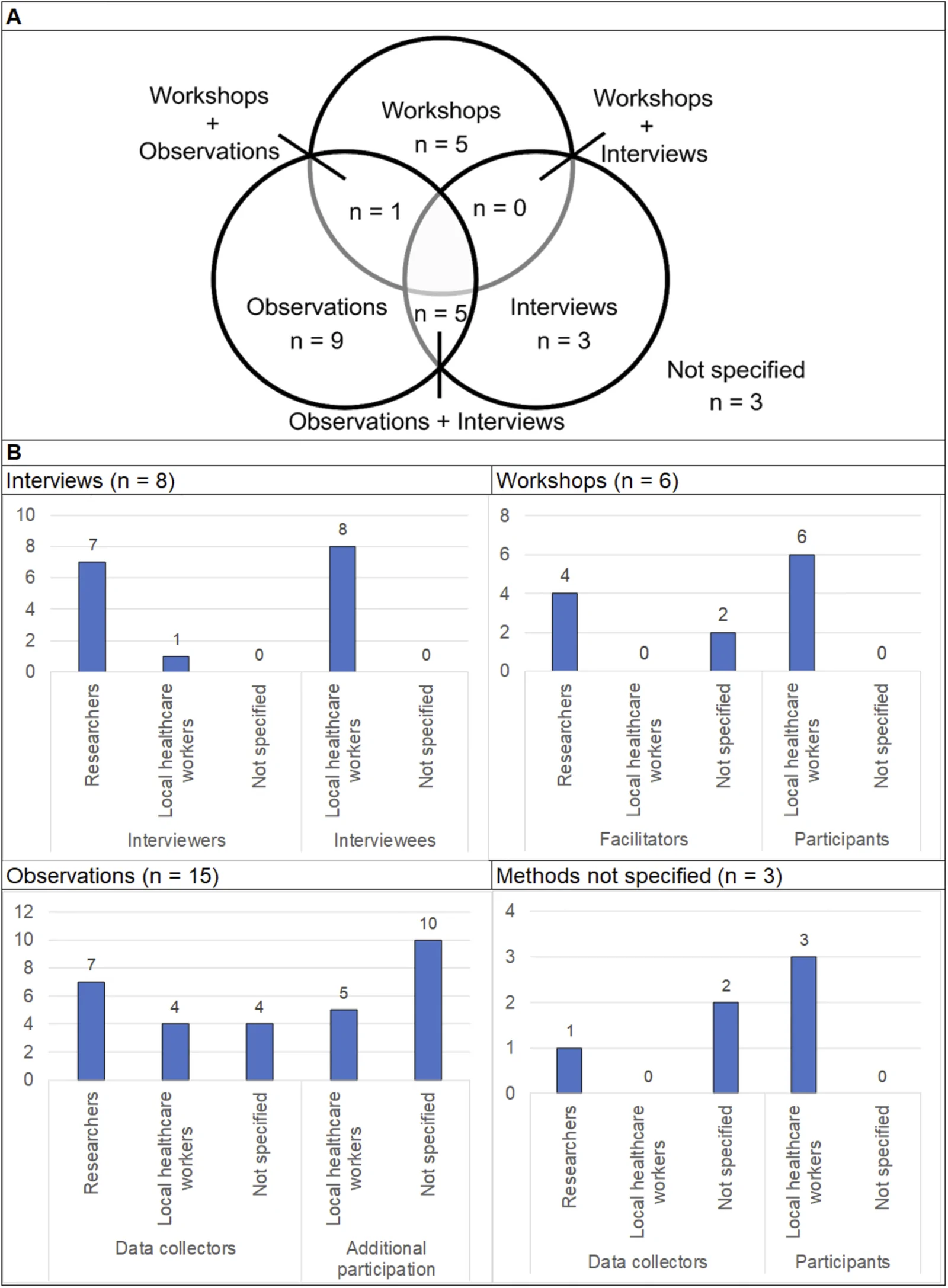
Methods used for process mapping data collection/Diagrams created with Microsoft Excel [58] and Microsoft PowerPoint [62]. **(A)** Methods applied for process mapping data collection. **(B)** Data gathering methods. (Low-income and lower-middle- income countries, 2014–2024).

During observations (n = 15; 58%), the researchers reported having gathered the data in seven cases [12, 36, 38, 39, 44, 48, 50]. In five cases (19%), the data collectors interacted with local healthcare workers while collecting the data through observations [35, 38, 39, 44, 46]. All studies justified these informal interactions during observation as a means of deepening understanding or clarifying unclear aspects of the observed workflow/patient flow. In the other ten cases (38%), additional interaction during data collection was not specified [12, 17, 21, 36, 37, 47, 42, 45, 48, 50].

Interviews were used in eight studies (31%), with data collected by the researchers in seven [35, 36, 38, 47, 48, 52, 54], see Figure 4B. In all eight studies (31%) using interviews, participants were local healthcare workers with expertise in their field and/or experience with the process under review.

In three studies (12%), the data collection methods were not described [34, 40, 51].

None of the included studies described the involvement of patient representatives during their data collection or at a later phase to generate the process map. Three studies (12%) involved patient representatives in other parts of their research, distinct from the PM activities [43, 45, 52].

### Phase 3: process map generation

Over half of the studies (n = 15; 58%) reported on generating a process map during or after data collection [12, 14, 17, 33, 35, 36, 38, 41–43, 45, 47, 48, 52, 53], but six of those failed to report any further detailed information [17, 36, 38, 42, 45, 48]. Among the studies (n = 6; 23%) that combined observations with workshops or interviews [35–38, 41, 48], five studies (19%) mentioned combining the data to generate the process map [35, 36, 38, 41, 48], yet lacked sufficient reporting details on how the data was combined.

### Phase 4: process map analysis

Analysis of the process maps was reported in 22 studies (85%), but only nine (35%) described the analysis in detail, focusing on identifying bottlenecks, inefficiencies, potential improvements in the process maps [12, 14, 21, 33, 41–44, 49]. Fishbone analysis, a commonly used method to identify root causes of a problem, was reported in three cases [21, 41, 49].

In six studies (23%), validation of the final process map was specifically sought through feedback from local healthcare workers [14, 33, 44, 47, 52, 53].

### Phase 5: take it forward

Taking action and implementing changes were reported in 27% (n = 7) of the studies, all of which were classified under the overarching objective “Process- and quality-improvement initiatives”. Another 69% (n = 18) highlighted processes or system challenges that required improvement. While these studies often suggested specific actions for improvement, they did not report to what extent the proposed changes were implemented. Only one study (4%) did not report on required improvements or action [34].

Of the 26 included studies, complete reporting of all variables was observed in only two studies [14, 33]. Eight studies had one missing variable [12, 36, 39, 41, 43, 47, 52, 53], while sixteen had multiple variables missing and lacked significant data [17, 21, 34, 35, 37, 38, 40, 42, 44–46, 48–51, 54].

## Discussion

This review examined PM in clinical healthcare settings in LLMICs, focusing on the extent of its application across geographies and clinical settings, intended purposes and implementation approaches.

### Versatility and adaptability of process mapping

Our findings demonstrate that PM has been conducted in diverse projects and initiatives in a number of LLMICs. PM was applied in a wide range of healthcare settings to map out different types of processes, showcasing its versatility and adaptability. This reflects its potential for widespread application in improving clinical processes and systems.

With 26 studies identified, this review indicates a marked increase in the use of PM in clinical healthcare settings in LLMICs over the last 5 years. A 2021 systematic review on PM [7], which included studies published up to 2019, reported only five studies from LLMICs out of 105 included. While this publication may have played a role in the increasing use of PM, other factors are likely to have contributed. For example, the high number of publications from India may reflect the influence of the WHO South-East Asian Regional Office’s 2020 publications which promoted the use of PM as a tool to understand and improve processes and systems in healthcare [63]. Despite this growth, uptake remains concentrated in a small number of countries, suggesting uneven uptake or reporting of PM across LLMICs.

### Process mapping phases

This review found that studies only partially applied the phases recommended in Antonacci et al.’s [7] conceptual framework. In particular, the “preparation” phase and the final “take it forward” phase were frequently underreported or omitted.

The finding that only a third of studies reported this final “take it forward” phase is particularly important, given its central role in translating findings into meaningful change. While underreporting may partly explain this, the frequent absence of documented action suggests that PM is often not carried through to implementation.

Two factors may contribute to this pattern. Firstly, PM was often reported as one component of a larger research project, where it may have functioned as an exploratory component rather than a tool for change. As a result, later PM phases (e.g. “take it forward”) may have been outside the study scope or deprioritised. Secondly, the fact that most PM studies were not locally led initiatives may also have limited progression to action, as local ownership is often required to identify, plan, operationalise and sustain improvements [64].

For practitioners considering using PM, these findings highlight the importance of approaching PM as more than an exploratory or descriptive exercise. To maximise its value, PM initiatives should explicitly plan (and document) all phases from the outset. To “take it forward”, this includes identifying relevant stakeholders and ensuring local leadership or co-leadership, and allocating time and resources to this phase.

### Use and reporting of methods

Most studies (65%) employed a single qualitative method to gather information for PM, with observations as the most prevalent, followed by workshops and interviews. While this may reflect resource constraints, it may limit the depth and completeness of the resulting process maps. Combining two or more methods can strengthen the completeness and validity of PM by taking advantage of the unique strength of each approach and allowing for triangulation [7, 8].

Reporting of methods was often inadequate, only 8% (n = 2) of all studies provided complete methodological reporting [14, 33]; 31% omitted only minor details [12, 36, 39, 41, 43, 47, 52, 53]; 35% lacked significant details [17, 34, 35, 41, 42, 45, 49, 51, 60]; and 12% did not report the method used to generate the PM [34, 51, 60]. An additional 13 studies were excluded from this review because, although they mentioned PM, they did not report on how it was conducted (see Supplementary Material 2). This lack of transparency limits both the credibility of individual studies and the ability to compare or build on findings across settings. These gaps demonstrate the need for more consistent and appropriate adherence to established research reporting guidelines.

These findings align with evidence of poor adherence to reporting guidelines in quality improvement research and implementation science [65–67].

Future researchers on PM initiatives should consider involving methodological experts [68] and using reporting guidelines to enhance the transparency and completeness of reporting. The choice of guideline should reflect the methodological approach of the study. For example, qualitative components of PM (e.g. interviews or workshops) may be reported using “Consolidated criteria for reporting qualitative research” (COREQ) guidelines [69, 70], while broader quality improvement initiatives may align better with SQUIRE or similar frameworks [71]. Where PM is used as a distinct methodological approach, the framework proposed by Antonacci et al. [7] should be used given its tailored guidance on documenting all phases of the PM process.

### Potential sources of bias in process mapping

The limited reporting of data collection methods and sampling during PM precluded a comprehensive assessment of potential biases affecting the respective process maps. The considerations below are conceptual in nature rather than derived from a systematic appraisal. They identify potential sources of bias associated with PM data collection methods to inform the design and reporting of future studies. During observational data collection, the Hawthorne effect [72, 73] may occur, where observed participants may alter their behaviour due to being observed. Five included studies reported direct contact with the workers who were directly involved in the observed process [35, 38, 39, 44, 46]. While such interaction can offer valuable insights, it should be weighed against the risk of altering participants’ behaviours [34, 35, 39, 74, 75].

Social desirability bias may have affected data collected during meetings, particularly when participants from different hierarchical levels were involved [76, 77]. Although understanding different viewpoints and diverse perspectives from different hierarchical levels is vital for a comprehensive understanding of processes [7], the potential impact of such a bias must be acknowledged and addressed, for example by separating into groups as done in a study in 2012 on PM in Ghana [13].

### Local involvement

Almost all studies involved local healthcare workers, yet researchers generally remained the leaders of the PM activities and only 15% [21, 37, 41, 46] reported local healthcare worker leadership. While the involvement of local stakeholders is valuable, the local leadership of PM activities is crucial for fostering ownership and ensuring long-term sustainability of the PM outcomes [78]. A notable example from a 2017 study in Ghana [14] highlighted the potential of locally led PM. While researchers facilitated the PM activity, the overall project was locally led with continuous collaboration between the researchers and local healthcare workers. The study reported that following the end of the study’s activity, “the local staff has adopted PM to aid in the design of several new initiatives” [14]. This case emphasises the importance of collaboration and local ownership in sustaining process improvement methods. Facilitating inclusive engagement, particularly across roles and organisational levels, can improve the validity of process maps and their relevance to the local context.

This findings are supported by broader literature which highlights that frontline-led initiatives are more likely to achieve lasting, contextually adapted outcomes [79, 80]. Further evidence indicates that shifting leadership and decision-making power to local actors is essential for improving research outcomes, accountability and sustainability [22, 80].

Therefore, future researchers, investigators, and funders of PM studies should prioritise establishing and sustaining local ownership from the planning phase of a study, to support long-term implementation and impact.

### Patient representation

A striking gap in patient participation was found, with none of the studies involving patients or patient representatives in the PM activities. Incorporating patient perspectives is essential for understanding patient flow and journeys in a holistic way. As reported by Trebble et al. [8], PM is an effective method for aligning care with patient-identified priorities; an objective which cannot be achieved without their direct input. Two studies cited feasibility as a barrier, particularly the need for translation into the local language [34, 53], but this could be easily overcome through locally led PM, eliminating the need for costly translation and making patient involvement more feasible. This interpretation is consistent with the WHO framework on integrated, people-centred health services [81]. This framework emphasises stronger patient involvement in designing and improving care. However, it acknowledges that patient involvement is less established in low-resource countries, which may further explain our findings [1, 82].

In recent years, patient and public involvement initiatives have risen across healthcare and research. However, reviews on patient involvement in quality improvement initiatives highlight that there is still a lack of evidence regarding how and when patient involvement is most useful. Moreover, these reviews emphasise the importance of tailored and context-specific involvement [83–85].

To ensure a holistic understanding of the process, it is recommended that patient involvement in PM initiatives should be systematically integrated. Similarly, researchers should incorporate a patient and public involvement approach into the design and implementation of their studies.

### Limitations

This scoping review has several limitations, mostly due to the scope and feasibility. Insufficient reporting of relevant information was a common issue in the included studies, which limited the completeness and interpretability of our findings. In addition, we restricted this review to peer-reviewed literature. Including grey literature may have added further depth through examples of PM from programme reports, but was not feasible given the global scope across 81 LLMICs. It is likely that the published literature underrepresents the actual use of PM, particularly where it is embedded in routine quality improvement activities and not perceived as research-oriented or worthy of publication. As a result, our findings may not reflect the full extent of PM implementation in LLMICs. Finally, although quality appraisal was not conducted, as is standard for scoping reviews, many included studies lacked sufficient methodological detail to assess rigour or reliability.

### Recommendations

This review recommends that clinical and non-clinical stakeholders involved in improvement initiatives consider PM as a valuable, adaptable tool, well-suited to strengthening processes in diverse clinical healthcare settings in LLMICs.

Future PM initiatives should consider all phases outlined by Antonacci [7] and triangulate multiple research methods where feasible, to enhance validity. Method selection should be carefully tailored to the specific type of process being mapped. Moreover, sampling strategies should be carefully considered to avoid potential sources of bias. Adherence to standardised reporting guidelines will improve quality, transparency and utility of PM outputs.

PM led or co-led by healthcare providers is especially encouraged, both to promote ownership and support sustainable improvements. Efforts should include diverse stakeholders’ perspectives, with prioritisation of patient involvement. Finally, greater attention must be paid to the final step of PM, “take it forward”, to ensure that PM leads to the implementation of tangible, contextually tailored, improvements in healthcare for all.

### Conclusion

This scoping review found that PM is an adaptable and increasingly used method in LLMICs, applied to a range of healthcare processes and purposes. However, our findings suggest that the potential of PM to support health system improvement is constrained by insufficient reporting transparency, limited stakeholder and patient engagement, and weak progression to implementation. Strengthening transparency, promoting locally led approaches, and ensuring inclusion of patient perspectives will be important to enhance the effectiveness and sustainability of PM in these settings.
