# Supplementary material for: Process mapping in clinical healthcare settings in low-income and lower-middle-income countries: a scoping review
Source: Public Health Rev. 2026 Oct 14;47:1609105. doi: 10.3389/phrs.2026.1609105 (PMC13713439; doi:10.3389/phrs.2026.1609105)
Supplement: Supplementary file 1 [file DataSheet1.PDF]

Supplementary File 1 : Search strategy

Database: Medline AND Embase

Platform: OVID

Data Run: 12.06.2024

Date range: 2014 – 2024

Limiters applied: Publication Date 2014 – 2024 /Language: English

Note: Publication Date only annual restriction: No possibility in the OVID database to limit to 01.01.2014 to 31.05.2024

Search strategy:

1. Concept: Process mapping

(((process OR journey OR patient experience OR care OR healthcare) adj3 (map*)) OR ((patient OR care OR healthcare) adj3 (journey OR pathway))).ti,ab.

2. Context I: Low- and lower-middle-income countries

(afghan* OR africa* OR algeria* OR "angola*or bangladesh*" OR benin* OR bhutan* OR bolivia* OR burkina* OR burkinese* OR burundi* OR "cabo verde*" OR "cape verde*" OR cambodia* OR cameroon* OR cameroun* OR chad* OR comoro* OR comore* OR comorian* OR congo* OR "cote d'ivoir*" OR "cote d' ivoir*" OR "cote divoir*" OR "cote d ivoir*" OR "ivory coast*" OR ivorian* OR djibouti* OR egypt* OR guinea* OR eritrea* OR eswatini* OR swaziland* OR swazi* OR swati* OR ethiopia* OR gambia* OR ghana* OR haiti* OR hondura* OR india* OR iran* OR jordan* OR kenya* OR korea* OR kyrgyz* OR kirgiz* OR kirghiz* OR laos OR lao OR laotian* OR lebanon* OR lebanese* OR lesotho* OR lesothan* OR lesothonian* OR liberia* OR madagasca* OR malawi* OR mali OR malian* OR mali's OR micronesia* OR kiribati* OR mauritania* OR mauritan* OR mongol* OR morocco* OR moroccan* OR mozambique* OR mozambican* OR myanmar* OR burma* OR burmese OR nepal* OR nicaragua* OR niger* OR pakistan* OR philippine* OR philipine* OR phillipine* OR phillippine* OR filipino* OR filipina* OR rwanda* OR rwandese OR ruanda* OR ruandese OR samoa* OR "sao tome*" OR santomean* OR senegal* OR "sierra leone*" OR "solomon island*" OR somali* OR "sri lanka*" OR sudan* OR syria* OR tajik* OR tadjik* OR tadzhik* OR tanzania* OR "timor leste*" OR "east timor*" OR timorese* OR togo OR togoles* OR togo's OR tunisia* OR uganda* OR ukrain* OR uzbek* OR vanuatu* OR vietnam* OR "viet nam*" OR yemen* OR zambia* OR zimbabwe* OR "arab countr*" OR "middle east*" OR "global south" OR sahara* OR subsahara* OR magreb* OR maghrib* OR "west indies*" OR caribbean* OR "central america*" OR "latin america*" OR "south america*" OR "asia central" OR "central asia*" OR "asia northern" OR "north asia*" OR "northern asia*" OR "asia southeastern" OR "southeastern asia*" OR "south eastern asia*" OR "southeast asia*" OR "south east asia*" OR "asia western" OR "west asia*" OR "western asia*" OR "europe eastern" OR "east europe*" OR "eastern europe*" OR "developing countr*" OR "developing nation*" OR "developing population*" OR "developing world" OR "less developed countr*" OR "less developed nation*" OR "less developed world" OR "lesser developed countr*" OR "lesser developed nation*" OR "lesser developed world" OR "under developed countr*" OR "under developed nation*" OR "under developed world" OR "underdeveloped countr*" OR "underdeveloped nation*" OR "underdeveloped world" OR "middle income countr*" OR "middle income nation*" OR "middle income population*" OR "low income countr*" OR "low income nation*" OR "low income population*" OR "lower income countr*" OR "lower income nation*" OR "lower income population*" OR "underserved countr*" OR "underserved nation*" OR "underserved population*" OR "under served population*" OR "under served nation*" OR "under served population*" OR "deprived countr*" OR "deprived population*" OR "high burden countr*" OR "high burden nation*" OR "countdown countr*" OR "countdown nation*" OR "poor countr*" OR "poor nation*" OR "poor population*" OR "poor world" OR "poorer countr*" OR "poorer nation*" OR "poorer population*" OR "poorer world" OR "developing econom*" OR "less developed econom*" OR "underdeveloped econom*" OR "under developed econom*" OR "middle income econom*" OR "low income econom*" OR "lower income econom*" OR "low gdp" OR "low gnp" OR "low gross domestic" OR "low gross national" OR "lower gdp" OR "lower gnp" OR "lower gross domestic" OR "lower gross national" OR lmic OR lmics OR "third world" OR "lami countr*" OR "transitional countr*" OR "emerging econom*" OR "emerging nation*" OR "low resource setting*" OR "resource limited setting*" OR "low resource setting*" OR "resource limited setting*" OR "resource constrained setting*" OR "central african republic*" OR "south sudan*" OR guinea-bissau* OR "republic of guinea-bissau*" OR nigeria* OR "papua new guinea*" OR "new guinea, papua*").ti,ab,hw,kf.

NOTE:*Source: Cochrane Effective Practice and Organisation of Care (EPOC) LMIC filters – February 2023* (1)*Major adaptation of search terms:Removal for LMIC countries classified by World bank (2) as well as removal names for countries / territories during the colonial period (irrelevant for year period 2014 – 2024) Manually added: "low resource setting" and "resource limited setting" and "resource constrained setting" as well as countries missing: Central African Republic, South Sudan, Guinea-Bissau, Nigeria and Papua New Guinea*

3. Context II: Clinical healthcare setting

Not applicable Database is healthcare setting

4. 1 AND 2

Result: Total: n = 1864 (Embase n = 1131 / Medline n = 733)

Database: CINAHL

Platform: CINAHL

Data Run: 12.06.2026

Date range: 01. 2014 – 05. 2024

Limiters applied: Publication Date 01. 2014 – 05. 2024 /Language: English / Removal: Apply equivalent subjects / Abstract available
Note: Publication date only annual restriction: No possibility in the CINAHL database to limit to 01.01.2014 to 31.05.2024

Search strategy:

1. Concept: Process mapping

((((TI process OR AB process) OR (TI journey OR AB journey) OR (TI "patient experience" OR AB "patient experience") OR (TI care OR AB care) OR (TI healthcare OR AB healthcare)) N3
((TI map* OR AB map*))) OR (((TI patient OR AB patient) OR (TI care OR AB care) OR (TI healthcare OR AB healthcar e)) N3 ((TI journey OR AB journey) OR (TI pathway OR AB pathway))))

2. Context I: Low- and lower-middle- income countries

(MH "Low and Middle Income Countries") OR (MH "Honduras") OR (MH "Nicaragua") OR (MH "Bolivia") OR (MH "Haiti") OR (MH "Tajikistan") OR (MH "Kyrgyzstan") OR (MH "Myanmar") OR (MH "Vietnam") OR (MH "Philippines") OR (MH "East Timor") OR (MH "Cambodia") OR (MH "Pakistan") OR (MH "Nepal") OR (MH "Sri Lanka") OR (MH "Afghanistan") OR (MH "Iran") OR (MH "Syria") OR (MH "Yemen") OR (MH "Mongolia") OR (MH "Low and Middle Income Countries") OR (MH "Algeria") OR (MH "Egypt") OR (MH "Morocco") OR (MH "Tunisia") OR (MH "Cameroon") OR (MH "Burundi") OR (MH "Central African Republic") OR (MH "Chad") OR (MH "Democratic Republic of the Congo") OR (MH "Equatorial Guinea") OR (MH "Congo") OR (MH "Djibouti") OR (MH "Eritrea") OR (MH "Ethiopia") OR (MH "Kenya") OR (MH "Rwanda") OR (MH "Somalia") OR (MH "Sudan") OR (MH "Tanzania") OR (MH "Angola") OR (MH "Zimbabwe") OR (MH "Malawi") OR (MH "Mozambique") OR (MH "Lesotho") OR (MH "Zambia") OR (MH "Benin") OR (MH "Burkina Faso") OR (MH "Cape Verde") OR (MH "Cote d'Ivoire") OR (MH "Gambia") OR (MH "Ghana") OR (MH "Guinea") OR (MH "Guinea-Bissau") OR (MH "Liberia") OR (MH "Mali") OR (MH "Mauritania") OR (MH "Niger") OR (MH "Nigeria") OR (MH "Senegal") OR (MH "Togo") OR (MH "Sierra Leone") OR (MH "Ukraine") OR (MH "India") OR (MH "Bangladesh") OR (MH "Laos") or (LMIC or LMICs or (low* N3 (income countr* or income nation*)) or Afghanistan OR Afghani OR Algeria* OR Angola* OR "Guinea-Bissau" OR Somalia OR Bangladesh* OR Belize OR Benin OR Bhutan or Bhutanese OR Bolivia* OR "Burkina Faso" OR Burundi OR "Central African Republic" OR "Cabo Verde" OR "Cape Verde" or Cambodia* OR Cameroon OR Chad OR Tchad OR Comoros OR Congo OR "Cote d Ivoire" OR "Ivory Coast" OR Djibouti OR Egypt* OR Eritrea* OR Eswatini OR Ethiopia* OR Gambia OR Ghana OR Guinea OR Haiti OR Honduras* OR India OR Iran OR Kenya or Kenyan OR Kiribati OR Kyrgyz OR Laos OR Lesotho OR Liberia OR Madagascar OR Malawi OR Mali OR Mauritania OR Micronesia OR Mongolia OR Morocco OR Mozambique OR Myanmar OR Nepal OR Nepalese OR Nicaragua* OR Niger OR Nigerien OR Nigeria* OR "North Korea" OR "Papua New Guinea" OR Pakistan OR Philippines OR Principe OR "Sierra Leone" OR "South Sudan" OR Sudan OR Syria OR Syrian OR Rwanda* OR Samoa* OR "Sao Tome" OR Senegal* OR "Solomon Islands"OR "Sri Lanka" OR Tanzania OR Tajikistan OR "Timor-Leste" OR Togo OR Tunisia OR Uganda OR Ukraine OR Uzbekistan OR Vanuatu OR Vietnam OR Yemen OR Zambia OR Zimbabwe OR OR Central African Republic OR Jordan OR Lebanon OR Papua New Guinea OR Burma OR Swaziland (TI "resource limited setting" OR AB "resource limited setting) OR (TI "low resource setting" OR AB "low resource setting") OR (TI "resource constrained setting" OR AB "resource constrained setting")

NOTE: Search term filter EPOC LMIC filter is not valid within CINAHL database, a similar filter was applied: Campbell, SM. A Filter to Identify Countries with Low or Lower Middle Income Economies in the EMBASE Database. Geoffrey & Robyn Sperber Health Sciences Library, University of Alberta. Rev. July 28, 2021 (2). Minor Adaptation: Removal of Venezuela, El Salvador and Belize and Indonesia, Bhutan, West Bank, Gaza. Manually added: “low resource setting” and “resource limited setting” and “resource constrained setting” as well as countries Central African Republic, Jordan, Lebanon, Papua New Guinea, Burma and Swaziland to assure accuracy with the other filter

3. Context II: Clinical healthcare setting
Not applicable Database is healthcare setting

4. 1 AND 2

Result: n = 433

Database: Web of Science Core Collection

Platform: WoS

Data Run: 12.06.2026

Date range: 01. 01. 2014 – 31. 05. 2024

Limiters applied**:** Publication Date 01. 01. 2014 – 31. 05. 2024 / Language: English /

Search strategy:

1. Concept: Process mapping

TS=(((process OR journey OR "patient experience" OR care OR healthcare) NEAR/3 (map*)) OR ((patient OR care OR healthcare) NEAR/3 (journey OR pathway)))

2. Context I: Low- and lower-middle- income countries

TS=((afghan* OR africa* OR algeria* OR "angola*or bangladesh*" OR benin* OR bhutan* OR bolivia* OR burkina* OR burkinese* OR burundi* OR "cabo verde*" OR "cape verde*" OR cambodia* OR cameroon* OR cameroun* OR chad* OR comoro* OR comore* OR comorian* OR congo* OR "cote d'ivoir*" OR "cote d' ivoir*" OR "cote divoir*" OR "cote d ivoir*" OR "ivory coast*" OR ivorian* OR djibouti* OR egypt* OR guinea* OR eritrea* OR eswatini* OR swaziland* OR swazi* OR swati* OR ethiopia* OR gambia* OR ghana* OR haiti* OR hondura* OR india* OR iran* OR jordan* OR kenya* OR korea* OR kyrgyz* OR kirgiz* OR kirghiz* OR laos OR lao OR laotian* OR lebanon* OR lebanese* OR lesotho* OR lesothan* OR lesothonian* OR liberia* OR madagasca* OR malawi* OR mali OR malian* OR mali's OR micronesia* OR kiribati* OR mauritania* OR mauritan* OR mongol* OR morocco* OR moroccan* OR mozambique* OR mozambican* OR myanmar* OR burma* OR burmese OR nepal* OR nicaragua* OR niger* OR pakistan* OR philippine* OR philipine* OR phillipine* OR phillippine* OR filipino* OR filipina* OR rwanda* OR randes OR ruanda* OR rwandese OR samoa* OR "sao tome*" OR santomean* OR senegal* OR "sierra leone*" OR "solomon island*" OR somali* OR "sri lanka*" OR sudan* OR syria* OR tajik* OR tadjik* OR tadzhik* OR tanzania* OR "timor leste*" OR "east timor*" OR timorese* OR togo OR togoles* OR togo's OR tunisia* OR uganda* OR ukrain* OR uzbek* OR vanuatu* OR vietnam* OR "viet nam*" OR yemen* OR zambia* OR zimbabwe* OR "arab countr*" OR "middle east*" OR "global south" OR sahara* OR subsahara* OR magreb* OR maghrib* OR "west indies*" OR caribbean* OR "central america*" OR "latin america*" OR "south america*" OR "asia central" OR "central asia*" OR "asia northern" OR "north asia*" OR "northern asia*" OR "asia southeastern" OR "southeastern asia*" OR "south eastern asia*" OR "southeast asia*" OR "south east asia*" OR "asia western" OR "west asia*" OR "western asia*" OR "europe eastern" OR "east europe*" OR "eastern europe*" OR "developing countr*" OR "developing nation*" OR "developing population*" OR "developing world" OR "less developed countr*" OR "less developed nation*" OR "less developed world" OR "lesser developed countr*" OR "lesser developed nation*" OR "lesser developed world" OR "under developed countr*" OR "under developed nation*" OR "under developed world" OR "underdeveloped countr*" OR "underdeveloped nation*" OR "underdeveloped world" OR "middle income countr*" OR "middle income nation*" OR "middle income population*" OR "low income countr*" OR "low income nation*" OR "low income population*" OR "lower income countr*" OR "lower income nation*" OR "lower income population*" OR "underserved countr*" OR "underserved nation*" OR "underserved population*" OR "under served population*" OR "under served nation*" OR "under served population*" OR "deprived countr*" OR "deprived population*" OR "high burden countr*" OR "high burden nation*" OR "countdown countr*" OR "countdown nation*" OR "poor countr*" OR "poor nation*" OR "poor population*" OR "poor world" OR "poorer countr*" OR "poorer nation*" OR "poorer population*" OR "poorer world" OR "developing econom*" OR "less developed econom*" OR "underdeveloped econom*" OR "under developed econom*" OR "middle income econom*" OR "low income econom*" OR "lower income econom*" OR "low gdp" OR "low gnp" OR "low gross domestic" OR "low gross national" OR "lower gdp" OR "lower gnp" OR "lower gross domestic" OR "lower gross national" OR lmic OR lmics OR "third world" OR "lami countr*" OR "transitional countr*" OR "emerging econom*" OR "emerging nation*" OR "low resource setting*" OR "resource limited setting*" OR "low resource setting*" OR "resource limited setting*" OR "resource

constrained setting*" OR "central african republic*" OR "south sudan*" OR guinea-bissau* OR "republic of guinea-bissau*" OR nigeria* OR "papua new guinea*" OR "new guinea, papua*"))

Note:

Source: Cochrane Effective Practice and Organisation of Care (EPOC) LMIC filters – February 2023 (1) Major adaptation of search terms:Removal for LMIC countries classified by World bank (3) as well as removal names for countries / territories during the colonial period (irrelevant for year period 2014 – 2024) Manually added: "low resource setting" and "resource limited setting" and "resource constrained setting" as well as countries missing: central African republic, South Sudan, Guinea-Bissau, Nigeria and Papua New Guinea

3. Context II: Clinical healthcare setting

TS=("health service*" OR "health sector" OR "primary health care" OR "PHC" OR "tertiary health care" OR "secondary health care" OR "health facilities" OR "health clinic*")

4. 1 AND 2 AND 3

Result: n= 324

Overall search results:

	Records:
MEDLINE:	n= 733
EMBASE:	n= 1131
CINAHL:	n= 433
WoS:	n= 324
Total before deduplication:	n= 2621
Duplicates removed:	n= 1051
Results after deduplication:	n= 1570

References:

1. Cochrane EPOC group. LMIC-filters (February 2023) [Internet] [Internet]. Zenodo. 2023 Jun 9;Zenodo. doi:10.5281/zenodo.8019937
2. Campbell S. A Filter to Identify Countries with Low or Lower Middle Income Economies in the EMBASE Database [Internet]. 2021 [cited 2025 Jan 22]. Low Middle Income Countries. Available from: https://docs.google.com/document/d/1pfn68ZvMEaNdbbmreh-1URWhQwKLTKrlx9FV7FAS4f4/edit?usp=embed_facebook
3. World Bank Group. World Bank country and lending groups [Internet]. 2024 [cited 2024 Apr 2]. Available from: https://datahelpdesk.worldbank.org/knowledgebase/articles/906519-world-bank-country-and-lending-groups
