# Supplementary material for: Process mapping in clinical healthcare settings in low-income and lower-middle-income countries: a scoping review
Source: Public Health Rev. 2026 Oct 14;47:1609105. doi: 10.3389/phrs.2026.1609105 (PMC13713439; doi:10.3389/phrs.2026.1609105)
Supplement: Supplementary file 2 [file DataSheet2.PDF]

Supplementary File 2 : Excluded Studies following full text screening

1. **Exclusion Criteria:** Wrong Concept : No description of their process map activities:

Total Wrong Concept no description of process map activities: Articles: n = 13

Authors	Titel Article	Name Journal	Country	Details Exclusion
Eastment et al. (2022)	Results of a cluster randomized trial testing the Systems Analysis and Improvement Approach to increase HIV testing in family planning clinics in Mombasa, Kenya	AIDS (London, England)	Kenya	Process mapping mentioned / does not describe process mapping acitivities / (process flow mapping)
Shidhaye et al. (2019)	Delivering a complex mental health intervention in low-resource settings: Lessons from the implementation of the PRIME mental healthcare plan in primary care in Sehore district, Madhya Pradesh, India	BJPsych Open	India	Process mapping mentioned / does not describe process mapping acitivities / (P. 3 + in supplement)
Snead et al. (2022)	'Mali Matters': reflections on a leadership and quality improvement project to improve blood transfusion safety at Maluti Adventist Hospital, Mapoteng, Lesotho	BMJ leader	Lesotho	Process mapping mentioned / does not describe process mapping acitivities (Existing pathway)
Jain et al. (2021)	Increasing adherence to plotting e- partograph: a quality improvement project in a rural maternity hospital in India	BMJ Open Quality	India	Process mapping mentioned / does not describe process mapping acitivities / (process flow chart)
Bhartia et al (2019)	Reducing delay in laboratory reports for outpatients from 16% to <3% at a non-profit hospital in New Delhi, India	BMJ Open Quality	India	Process mapping mentioned / does not describe process mapping acitivities / (process map waiting time)
Ranade et al. (2024)	Quality improvement initiative: improving obstetric triaging practices in a rural maternal hospital in central India	BMJ Open Quality	India	Process mapping mentioned / does not describe process mapping acitivities
Sadasivam et al. (2024)	Improving door-to-analgesia timing in musculoskeletal injuries in an academic emergency department in India: a quality improvement project	BMJ Open Quality	India	Process mapping mentioned / does not describe process mapping acitivities
Adane et al. (2025)	The revenue generated from clinical chemistry and hematology laboratory services as determined using activity-based costing (ABC) model	Cost Effectiveness and Resource Allocation : C/E	Ethiopia	Process mapping mentioned / does not describe process mapping acitivities / (Page 3)
Ouasrhir et al. (2020)	Use of a prospective risk analysis method to improve the safety of healthcare workers in oncology hospitals during the COVID-19 spread among cancer patients	European Journal of Oncology Pharmacy	Morocco	Process mapping mentioned / does not describe process mapping acitivities
Eastment et al. (2023)	Results of a cluster randomized trial testing the Systems Analysis and Improvement Approach to increase cervical cancer screening in family planning clinics in Mombasa County, Kenya	Implementation Science : IS	Kenya	Process mapping mentioned / does not describe process mapping acitivities / (process flow mapping)
Viswanath et al. (2021)	Organising Home-based Palliative Care Services: A Quality Improvement project at the Homi Bhabha Cancer Hospital and Research Centre, Vishakhapatnam	Indian Journal of Palliative Care	India	Process mapping mentioned / does not describe process mapping acitivities / (too short description under visualisation) /
Balakrishna et al. (2023)	Initiation of Palliative Care Referral from the Intensive Care Unit for Advanced Stage Metastatic Cancer Patients: A Quality Improvement Process from a Tertiary Referral Cancer Institute from South India	Indian Journal of Palliative Care	India	Process mapping mentioned / does not describe process mapping acitivities / very shortly ("gemma walk")
Forrester et al. (2021)	Clean Cut (adaptive, multimodal surgical infection prevention programme) for low-resource settings: A prospective quality improvement study	The British Journal of Surgery	Ethiopia	Process mapping mentioned / does not describe process mapping acitivities

2. **Exclusion Criteria:** Wrong Concept: Not process mapping

Total: Wrong Concept : no process mapping: Articles: n = 6

Authors	Titel Article	Name Journal	Country	Details Exclusion
Acacio-Claro et al (2022)	A Micro-analysis Approach in Understanding Electronic Medical Record Usage in Rural Communities: Comparison of Frequency of Use on Performance Before and During the COVID-19 Pandemic	Procedia Computer Science	Phillipines	Process mining, not process mapping
de Guzman et al (2022)	Patient waiting time analysis for elective gynecologic surgeries in a tertiary training hospital in the Philippines: A retrospective cross-sectional study	Annals of Medicine and Surgery	Phillipines	Mapping out processes, not process mapping
Engel et al. (2015)	Point-of-care testing in India: missed opportunities to realize the true potential of point-of-care testing programs.	BMC Health Services Research	India	Mapped out process of setting, not process mapping
Itanyi et al. (2023)	Rapid implementation mapping to identify implementation determinants and strategies for cervical cancer control in Nigeria	Frontiers in Public Health	Nigeria	Implementation mapping, not process mapping
Okonofua et al. (2018)	Where do delays occur when women receive antenatal care? A client flow multisite study in four health facilities in Nigeria	Ghana Medical Journal	Nigeria	Time flow, not process mapping
Tufail et al. (2022)	DMAIC methodology for achieving public satisfaction with health departments in various districts of Punjab and optimizing CT scan patient load in urban city hospitals	AIMS Public Health	Pakistan	Costumer value mapping, not process mapping

3. **Exclusion Criteria:** Wrong Source: Conference Abstract

Total Wrong Source: Conference Abstract: Articles: n= 23

Authors	Titel Article	Name Journal / Conference Paper	Country	Details Exclusion
Ahmed et al (2024)	Implementation and execution of integrated point of care testing program in three secondary hospitals across Karachi-a lean management approach	Clinica Chimica Acta	Pakistan	Conference Abstract
Bennani et al. (2018)	Use of a risk analysis method to improve safety to the chemotherapy circuit in hospital	European Journal of Oncology Pharmacy	Morocco	Conference Abstract
Caldwell et al. (2014)	Process improvement: A valuable tool for health-system strengthening in developing countries	Annals of Global Health	Ghana	Conference Abstract
Chang et al. (2016)	Utilizing process map-driven protocols as educational tools: Developing hypertension protocols for management of preeclampsia in Botswana	Annals of Global Health	Botswana	Conference Abstract
Chopra et al. (2022)	Implementation case study of image guided adaptive high dose rate brachytherapy for cervical cancer: workflow impact analysis of upgrading to image based brachytherapy within National Cancer Grid of India cervix cancer resource stratified guidelines	International Journal of Gynecologic Cancer	India	Conference Abstract
Emem-Chioma et al. (2020)	USE OF POINT OF CARE CREATININE FOR EARLY DETECTION AND MANAGEMENT OF COMMUNITY ACQUIRED ACUTE KIDNEY INJURY-STUDY DESIGN	Kidney International Reports	Nigeria	Conference Abstract
Fara et al. (2018)	A resident-driven intervention to decrease doorto- needle time and increase resident satisfaction in a resource-limited setting	American Heart Association	Not specified	Conference Abstract
Forrester et al. (2017)	A who surgical safety checklist-based infection prevention program in ethiopia: Using process mapping to identify barriers for implementation	Annals of Global Health	Ethiopia	Conference Abstract
Forresteret al. (2018)	Scoring System to Aid Implementation of a Surgical Infection Prevention Quality Improvement Program in Resource-Constrained Settings	Journal of the American College of Surgeons	Ethiopia	Conference Abstract
Gebeyehu et al. (2021)	Applying a quality improvement framework to increase operating room efficiency in tikur anbessa specialized Hospital, Ethiopia	Society for the Advancement of Patient Blood Management 2021 Scientific Abstracts	Ethiopia	Conference Abstract
Kabajaasi et al. (2022)	PEDIATRIC DISCHARGES IN UGANDA: DEVELOPMENT OF A DISCHARGE MODEL OF CARE	Pediatric Critical Care Medicine	Uganda	Conference Abstract
Kausar et al. (2024)	Mapping local laboratory interface codes to LOINC using RELMA: A team-based approach	Clinica Chimica Acta	Pakistan	Conference Abstract
Mandigo et al. (2015)	A time-driven activity-based costing model to improve health-care resource use in Mirebalais, Haiti.	The Lancet Commission on Global Surgery Abstracts	Haiti	Conference Abstract
Mintor et al. (2017)	Improving the diagnosis and early follow-up of children with sickle cell disease born in mirebalais haiti	Blood- American Society of Hematology	Haiti	Conference Abstract
Mokokwe et al. (2018)	First Things First: Adopting a Holistic, Needs-Driven Approach to Improving the Quality of Routinely Collected Data	Journal of Global Oncology	Botswana	Conference Abstract
Saeed et al. (2021)	Joint pulmonary-rheumatology clinic in lowresource Settings: A one year experience	Thorax	Pakistan	Conference Abstract
Satija et al. (2020)	A Quality Improvement Project to Provide Early Referral to Palliative Care Clinic for Advanced Oral Cancer Patients (TH341A)	Journal of Pain and Symptom Management	India	Conference Abstract
Soliman et al. (2017)	The delivery of value-based healthcare for children with osteosarcoma at Children's Cancer Hospital Egypt	Journal of Clinical Oncology	Egypt	Conference Abstract

Torzsok et al. (2018)	Time-driven activity-based costing for competing treatments using different technology solutions	Not specified	Not specified	Conference Abstract
Vadera et al. (2023)	The journey From Conceptualizing, Designing, and Implementing Our Integrated Smart Core Lab at Kokilaben Dhirubhai Ambani Hospital (KDAH)	Clinical Chemistry	India	Conference Abstract
Vemanamandhi et al. (2020)	Problem solving using the a3 methodology for colposcopy	International Journal of Gynecologic Cancer	India	Conference Abstract
Venkatesh et al. (2018)	Radiation exposure in Nicu	Quality Improvement RCPCH, Abstracts of the Annual Conference	India	Conference Abstract
Forrester et al. (2018)	Developing operating system process maps for surgical infection prevention: A tool to improve perioperative standards in low-and middle-income countries	Journal of the American College of Surgeons	Ethiopia	Conference Abstract

4. **Exclusion Criteria**: Wrong Source: Study Protocol

Total Wrong Source: Study Protocol: Article n = 6

**Authors**	**Titel Article**	**Name Journal**	**Country**	**Details Exclusion**
Gimbel et al. (2020)	Systems analysis and improvement approach to optimize the hypertension diagnosis and care cascade for PLHIV individuals (SAIA-HTN): a hybrid type III cluster randomized trial	Implementation Science IS	Mozambique	Study protocol
Mammo et al. (2020)	Evaluation of an adaptive, multimodal intervention to reduce postoperative infections following cesarean delivery in Ethiopia: study protocol of the CLEAN-CS cluster-randomized stepped wedge interventional trial	Trials	Ethiopia	Study protocol
Nessle et al. (2023)	Barriers to and facilitators of effective management of fever episodes in hospitalised Kenyan children with cancer: protocol for convergent mixed methods study	BMJ Open	Kenya	Study protocol
Rashan et al. (2023)	Mixed methods study protocol for combining stakeholder-led rapid evaluation with near real-time continuous registry data to facilitate evaluations of quality of care in intensive care units	Wellcome Open Research	Kenya, India, (Malaysia), Nepal, Pakistan, (South Africa), Uganda and Vietnam	Study protocol
Sherr et al. (2019)	Scaling-up the Systems Analysis and Improvement Approach for prevention of mother-to-child HIV transmission in Mozambique (SAIA-SCALE): a steppedwedge cluster randomized trial	Implementation Science	Mozambique	Study protocol
Harrison et al. (2021)	A mobile cesarean birth center as a solution to improve access to surgical birth in rural Ethiopia: a mixed methods research protocol	Pilot and Feasibility Studies	Ethiopia	Study protocol

5. **Exclusion Criteria**: Wrong Publish Time

Total Wrong Publish Time: Article n = 1

Authors	Titel Article	Name Journal	Country	Exclusion Details
Mbakaya et al. (Preprint 2024)	A Human Factors Approach to Childhood Pneumonia A qualitative study in Malawian primary care	Primary Care Research	Malawi	Wrong Publish Time

6. **Exclusion Criteria:** Language: French

Total Wrong Language: Article n = 1

Authors	Titel Article	Name Journal	Country	Exclusion Details
Tlijani et al. (2023)	Mapping of the risk management of the circuit of drugs derived from stable human blood in a Tunisian university hospital	Le Pharmacien Clinicien	Tunisia	Wrong Language
