# Supplementary material for: Process mapping in clinical healthcare settings in low-income and lower-middle-income countries: a scoping review
Source: Public Health Rev. 2026 Oct 14;47:1609105. doi: 10.3389/phrs.2026.1609105 (PMC13713439; doi:10.3389/phrs.2026.1609105)
Supplement: Supplementary file 3 [file DataSheet3.PDF]

Supplementary File 3: Data extraction sheet

Authors	Titel Article	Research Method	Name Jour-nal	Country study conducted	Health system level (Multiple responses possible)	Year/s of data collection	Health care setting/s (Multiple responses possible)	Clinical health speciality (Multiple responses possible)	Overarching aim
Gopinath et al. (2022)	Strengthening emergency care systems to improve patient care for ST-elevation myocardial infarction (STEMI) at a high-volume tertiary care centre in India	Multi method	BMJ Open Qual	India	Tertiary healthcare	2018 - 2019	Emergency Room	Cardiovascular diseases	Process- and quality improvement initiatives - PDSA
Kabajaasi et al. (2023)	Transitions from hospital to home: A mixed methods study to evaluate pediatric discharges in Uganda	Multi method	PLOS Glob Public Health	Uganda	Secondary healthcare and Tertiary healthcare	2020 - 2021	Outpatient and Inpatient	Child health (Pediatrics)	Health systems process analysis and assessment
Forrester et al. (2018)	Developing Process Maps as a Tool for a Surgical Infection Prevention Quality Improvement Initiative in Resource-Constrained Settings	Qualitative	J Am Coll Surg	Ethiopia	Tertiary healthcare	2016 - 2017	Inpatient and OT	Surgical procedures	Process- and quality improvement initiatives - PDSA
Ofosu B. et al. (2021)	Assessing the functionality of an emergency obstetric referral system and continuum of care among public healthcare facilities in a low resource setting: an application of process mapping approach	Qualitative	BMC Health Services Research	Ghana	Primary healthcare, Secondary healthcare, Tertiary healthcare	2019	Emergency Room and Outpatient	Maternal health	Health systems process analysis and assessment
Mula et al. (2018)	Assessment of factors that influence timely administration of initial antibiotic dose using collaborative process mapping at a referral hospital in Malawi: a case study of pneumonia patients	Multi method	BMC Infect Dis	Malawi	Tertiary healthcare	Not specified	Emergency Room	Infectious diseases	Process- and quality improvement initiatives - Solely Process Assessment

Villegas et al. (2021)	Task-shifting to optimize outpatient neurological care in Zambia	Multi method	Hum Resour Health	Zambia	Tertiary healthcare	2018	Outpatient	Neurological diseases	Process- and quality improvement initiatives - Others
Whitaker et al. (2023)	Novel application of multi-facility process map analysis for rapid injury care health system assessment in Northern Malawi	Qualitative	BMJ Open	Malawi	Primary healthcare	2019	Not specified	Ill-defined injuries / accidents	Health systems process analysis and assessment
Bebbington et al. (2023)	Establishing Self-Harm Registers: The Role of Process Mapping to Improve Quality of Surveillance Data Globally	Qualitative	Int J Environ Res Public Health	India	Tertiary healthcare	2019 - 2020	Not specified	Psychological health	Health systems tools design, implementation and evaluation
Aaronson et al. (2017)	Mapping the process of emergency care at a teaching hospital in Ghana	Qualitative	Health care	Ghana	Tertiary healthcare	Not specified	Emergency Room	Emergency Care Treatment	Process improvement / QI improvement - LEAN
Anjalee et al. (2021)	Application of failure mode and effects analysis (FMEA) to improve medication safety in the dispensing process – a study at a teaching hospital, Sri Lanka	Qualitative	BMC Public Health	Sri Lanka	Tertiary healthcare	2018	Outpatient	Other	Process- and quality improvement initiatives - FMEA
Bebbington et al. (2024)	Development of an electronic burns register: Digitisation of routinely collected hospital data for global burns surveillance	Multi method	Burns	India	Tertiary healthcare	2019 - 2020 (2016-2022 data registry)	Outpatient and Inpatient	Ill-defined injuries / accidents (Burns)	Health systems tools design, implementation and evaluation (digital)
Chirenda et al. (2021)	A feasibility study using time-driven activity-based costing as a management tool for provider cost estimation: lessons from the national TB control program in Zimbabwe in 2018	Multi method	BMC Health Serv Res	Zimbabwe	Primary healthcare and Secondary healthcare	2018 - 2019	Multiple (= / > 3 settings) No specification possible	Infectious diseases	Costing - TBABC

Drew et al. (2016)	Impact of patients' healthcare payment methods on hospital discharge process: evidence from India	Multi method	Int J Health Plann Manag e	India	Tertiary healthcare	2014	Inpatient	Not specified	Process- and quality improvement initiatives - Others
Gimbel et al. (2014)	The prevention of mother-to-child transmission of HIV cascade analysis tool: supporting health managers to improve facility-level service delivery	Multi method	BMC Res Notes	Mozambique	Not specified	2013	Multiple (= / > 3 settings) No specification possible	Infectious diseases and Maternal / Child Health	Health systems tools design, implementation and evaluation
Khan et al. (2019)	Time-driven activity-based costing of total knee replacements in Karachi, Pakistan	Multi method	BMJ Open	Pakistan	Tertiary healthcare	2015 - 2016	Multiple (= / > 3 settings) No specification possible	Surgical procedures	Costing - TBABC
Sunilkumar et al. (2021)	"Relax City Homecare team; protocols are ready for you" – A Quality Improvement Project Conducted on Behalf of the City Homecare Unit Team at the Trivandrum Institute of Palliative Sciences	Multi method	Indian J Palliat Care	India	Not specified	Not specifie d	Other (Homecare)	Other	Process- and quality improvement initiatives - Others
Haroon et al. (2019)	Patient flow and waiting time in emergency department of tertiary health care hsopitals of khyber pakhtunkhwa	Multi method	J Mech Contin Math Sci	Pakistan	Tertiary healthcare	Not specifie d	Emergency Room	Emergency Care Treatment	Health systems process analysis and assessment
Kefyalew et al. (2023)	Reducing the length of stay of cardiac patients in the Adult Emergency department by using a Quality improvement framework	Multi method	Int Emerg Nurs	Ethiopia	Other	2021 - 2022	Emergency Room	Cardiovascular diseases	Process- and quality improvement initiatives - PDSA
McBain et al. (2023)	Costing of HIV services, Uganda and United Republic of Tanzania	Multi method	Bull World Health Organ	Tanzania / Uganda	Not specified	2020 - 2021	Multiple (= / > 3 settings) No specification possible	Infectious diseases	Costing - TBABC

Nguyen et al. (2023)	Mapping patient pathways and understanding clinical decision-making in dengue management to inform the development of digital health tools	Qualitative	BMC Med Inform Decis Mak	Vietnam	Tertiary healthcare	2020 - 2022	Emergency Room and Outpatient and Inpatient	Infectious diseases	Health system tools design / implementation / evaluation (digital)
Robertson et al. 2018)	IMCI and ETAT integration at a primary healthcare facility in Malawi: a human factors approach	Qualitative	BMC Health Servic es Resear ch	Malawi	Primary healthcare	Not specifie d	Outpatient	Child health (Pediatrics)	Process- and quality improvement initiatives - Others
Smiley et al. (2019)	Safety Culture and Perioperative Quality at the Volta River Authority Hospital in Akosombo, Ghana	Multi method	World J Surg	Ghana	Secondary healthcare	2017	OT	Surgical procedures	Process- and quality improvement initiatives - Others
Suwedi-Kapesa et al. (2024)	Process Evaluation of Services for HIV-Infected Post-Partum Women and HIV-Exposed Infants in Primary Health Care Blantyre Malawi	Multi method	Health Serv Insight s	Malawi	Primary healthcare	2019 - 2020 (2018 data)	Outpatient	Infectious diseases and Mother/Child Health	Health systems process analysis and assessment
Varghese et al. (2020)	Improving the uptake of newborn screening for endocrinopathies and inborn errors of metabolism: A quality improvement initiative.	Quantitative	J Neonat al Nurs	India	Tertiary healthcare	Not specifie d	Inpatient	Child health	Process- and quality improvement initiatives - PDSA
Whitaker et al. (2024)	Health system assessment for access to care after injury in low- or middle-income countries: A mixed methods study from Northern Malawi	Multi method	PLOS Medici ne	Malawi	Not specified	2019 - 2020	Not specified	Ill-defined injuries / accidents	Health systems process analysis and assessment
Zouladeny et al. (2015)	Epidemiologic and Clinical Profiles of Breast Diseases in Niger	Multi method	Int J Cancer Oncol	Niger	Secondary healthcare and Tertiary healthcare	2013 (2010 registry data)	Outpatient and Inpatient	Other	Health systems process analysis and assessment

Extraction Sheet (continued)

Authors	Type of processes being observed	Methods used for PM data collection	Prep-aration	Data and information gathering	Local HCW involvement	Patient representatives	Process map generation	Process map analysis	Validation final pm with local	Take it forward	Reporting and Documentation
Gopinath et al. (2022)	Patient flow in a specific clinical setting	Observation & Workshops	Yes	See separate Table Data collection Workshop / Observation	yes	No	Yes, during meetings	Yes, during meeting	Not specified but process map generated with local healthcare workers	Yes, implemented	Only one reporting variable missing - facilitator of pm exercise missing
Kabajaasi et al. (2023)	Workflow in a clinical setting	Workshops	Not specified	See separate Table Data collection Workshop	yes	No, but in other methods involved	Yes, during meetings	Yes, during meeting	Not specified but process map generated with local healthcare workers	No, but identified process or system challenges for improvement	Only one reporting variable missing - preparation missing
Forrester et al. (2018)	Workflow in a clinical setting	Observation	Yes	See separate Table Data collection Observation	yes	No	Not specified	Yes	Not specified but process map generated with local healthcare workers	Yes, implemented	Only one reporting variable missing- Process map generation missing
Ofosu B. et al. (2021)	Patient journey through a health system	Interviews	Not specified	See separate Table Data collection not specified	yes	No	Yes, after interviews	Yes, after interviews	Yes	No, but identified process or system challenges for improvement	Only one reporting variable missing- preparation missing
Mula et al. (2018)	Patient flow in a specific clinical setting	Observation	Yes	See separate Table Data collection Observation	not specified	No	Yes, during observation	Yes, after meetings	Not specified but process map generated with local healthcare workers	Yes, implemented	Only one reporting variable missing- local involvement missing

Villegas et al. (2021)	Patient flow in a specific clinical setting	Interviews	Not specified	See separate Table Data collection Interviews	yes	No but in another method	Yes, after interviews	Yes	Yes	No, but identified process or system challenges for improvement	Only one reporting variable missing- Preparation missing
Whitaker et al. (2023)	Patient journey through a health system	Workshops	Yes	See separate Table Data collection Workshop	yes	No	Yes, during meetings	Yes, after meeting	Yes	No, but identified process or system challenges for improvement	Only one reporting variable missing - health care setting missing
Bebbington et al. (2023)	Workflow within a clinical setting	Observation & Interviews	Yes	See separate Table Data collection Observation and Workshop	yes	No	Yes, but unclear how	Yes	Not specified	No, but identified process or system challenges for improvement	Only one reporting variable missing- Unclear Map generation specification
Aaronson et al. (2017)	Patient flow in a specific clinical setting	Workshops	Yes	See separate Table Data collection Workshop	yes	No	Yes, during meetings	Yes, during meeting	Yes	Yes, implemented	Complete
Anjalee et al. (2021)	Workflow in a clinical setting	Workshops	Yes	See separate Table Data collection Workshop	yes	No	Yes, during meetings	Yes, during meeting	Yes	Yes, implemented	Complete
Bebbington et al. (2024)	Workflow in a clinical setting	Observation & Interviews	Not specified	See separate Table Data collection Observation and Interviews	yes	No	Yes, after interviews	yes, after interviews	Not specified	No, but identified process or system challenges for improvement	Not complete- multiple variables missing
Chirenda et al. (2021)	Patient journey through a health system	Observation & Interviews	Not specified	See separate Table Data collection observation and Interviews	yes	No	Not specified	Yes	Not specified	No, but identified process or system challenges for improvement	Not complete- multiple variables missing

Drew et al. (2016)	Workflow in a clinical setting	Observation & Interviews	Not specified	See separate Table Data collection Obesrvation and Interviews	yes	No	Yes, but unclear how	Yes	Not specified	No, but identified process or system challenges for improvement	Not complete- multiple variables missing
Gimbel et al. (2014)	Patient flow in a specific clinical setting	Not specified	Not specified	See separate Table Data collection not specified	yes	No	Not specified	Not specified	Not specified but process map generated with local healthcare workers	No, but identified process or system challenges for improvement	Not complete- multiple variables missing
Khan et al. (2019)	Patient flow in a specific clinical setting	Observation	Not specified	See separate Table Data collection Observation	yes	No	Not specified	Yes	Yes with meetings (Local healthcare workers and management)	No, but identified process or system challenges for improvement	Not complete- multiple variables missing
Sunilkumar et al. (2021)	Workflow in a clinical setting	Workshops	Not specified	See separate Table Data collection Workshops	yes	No	Not specified	Yes	Not specified but process map generated with local healthcare workers	No, but identified process or system challenges for improvement	Not complete- multiple variables missing
Haroon et al. (2019)	Patient flow in a specific clinical setting	Observation	Yes	See separate Table Data collection Obesrvation	not specified	No	Yes, but unclear how	Yes	Not specified	No, but identified process or system challenges for improvement	Not complete- multiple variables missing
Kefyalew et al. (2023)	Patient flow in a specific clinical setting	Observation	Not specified	See separate Table Data collection Observation	yes	No	Not specified	Yes	Not specified but process map generated with local healthcare workers	Yes, implemented	Not complete- multiple variables missing

McBain et al. (2023)	Patient flow in a specific clinical setting	Observation	Yes	See separate Table Data collection Observation	not specified	No but in another method	Yes, but unclear how	Yes	Not specified	No, but identified process or system challenges for improvement	Not complete-multiple variables missing
Nguyen et al. (2023)	Patient flow in a specific clinical setting	Observation	Not specified	See separate Table Data collection Observation	yes	No	Not specified	Yes	Not specified but process map generated with local healthcare workers	No, but identified process or system challenges for improvement	Not complete-multiple variables missing
Robertson et al. 2018)	Patient flow in a specific clinical setting	Observation & Interviews	Not specified	See separate Table Data collection Observation and Interviews	yes	No	Yes, but unclear how	Yes	Not specified	No, but identified process or system challenges for improvement	Not complete-multiple variables missing
Smiley et al. (2019)	Workflow in a clinical setting	Observation	Not specified	See separate Table Data collection Observation	not specified	No	Yes, but unclear how	Yes	Not specified	No, but identified process or system challenges for improvement	Not complete-multiple variables missing
Suwedi-Kapesa et al. (2024)	Patient flow in a specific clinical setting	Observation	Not specified	See separate Table Data collection Observation	not specified	No	Not specified	yes, after interviews	Not specified	No, but identified process or system challenges for improvement	Not complete-multiple variables missing
Varghese et al. (2020)	Workflow in a clinical setting	Not specified	Not specified	See separate Table Data collection not specified	yes	No	Not specified	Not specified	Not specified	Yes, implemented	Not complete-multiple variables missing
Whitaker et al. (2024)	Patient journey through a health system	Not specified	Not specified	See separate Table Data collection not specified	yes	Not specified	Not specified	Not specified	Not specified	Not specified	Not complete-multiple variables missing

Zouladeny et al. (2015)	Patient journey through a health system	Interviews	Not specified	See separate Table Data collection not specified	yes	No	Not specified	Not specified	Not specified	No, but identified process or system challenges for improvement	Not complete- multiple variables missing

**Process mapping data collection: Workshops**			
**Author**	**Process mapping methodology**	**Workshops: Who facilitated in the pm workshop-exercise?**	**Meetings: Who participated in the pm workshop- exercise?**
Aaronson et al. (2017)	**Workshops**	Researchers	Local healthcare workers
Anjalee et al. (2021)	**Workshops**	Researchers	Local healthcare workers
Gopinath et al. (2022)	**Workshops** (and Observation)	Not specified	Local healthcare workers
Kabajaasi et al. (2023)	**Workshops**	Researchers	Local healthcare workers
Sunilkumar et al. (2021)	**Workshops**	Not specified	Local healthcare workers
Whitaker et al. (2023)	**Workshops**	Researchers	Local healthcare workers

**Process mapping data collection: Observation**			
**Author**	**Process mapping methodology**	**Data collection: Who collected the data during observation**	**Data collection: Additional contribution during observation?**
Bebbington et al. (2023)	**Observation** (and Interviews)	Researcher	Not specified
Bebbington et al. (2024)	**Observation** (and Interviews)	Not specified	Local healthcare workers (prompt further questions during observation when unclarity)
Chirenda et al. (2021)	**Observation** (and Interviews)	Local healthcare workers	Not specified
Drew et al. (2016)	**Observation** (and Interviews)	Researcher	Local healthcare worker (staff was asked questions if the work which was observed was unclear)
Forrester et al. (2018)	**Observation**	Researcher	Local healthcare workers (short informal interviews personell directly responsible for a particular step during observation)
Gopinath et al. (2022)	**Observation** (and Workshops)	Local healthcare workers as researchers	Not specified
Haroon et al. (2019)	**Observation**	Not specified	Not specified
Kefyalew et al. (2023)	**Observation**	Local healthcare workers	Not specified
Khan et al. (2019)	**Observation**	Researcher	Local healthcare workers (iterative discussions to understand the workflow for patient better)
McBain et al. (2023)	**Observation**	Not specified	Not specified
Mula et al. (2018)	**Observation**	Researcher	Not specified
Nguyen et al. (2023)	**Observation**	Local healthcare workers as researchers	Local healthcare workers (informal discussion with healthcare providers during observation)
Robertson et al. (2018)	**Observation** (and Interviews)	Researcher	Not specified
Smiley et al. (2019)	**Observation**	Not specified	Not specified
Suwedi- Kapesa et al. (2024)	**Observation**	Researcher	Not specified

**Process mapping: Data collection Interviews**			
**Author**	**Process mapping methodology**	**Data collection: Who conducted the interviews?**	**Data collection: Who were interviewed**
Bebbington et al. (2023)	**Interviews** (and Observation)	Researcher	Local healthcare workers
Bebbington et al. (2024)	**Interviews** (and Observation)	Researcher	Local healthcare workers
Chirenda et al. (2021)	**Interviews** (and Observation)	Local healthcare workers (trained beforehand)	Local healthcare workers
Drew et al. (2016)	**Interviews** (and Observation)	Researcher	Local healthcare workers
Ofosu et al. (2021)	**Interviews**	Researcher	Local healthcare workers
Robertson et al. (2018)	**Interviews** (and Observation)	Researcher	Local healthcare workers
Villegas et al. (2021)	**Interviews**	Researcher	Local healthcare workers
Zouladeny et al. (2015)	**Interviews**	Researcher	Local healthcare workers

**Process mapping: Data collection: not specified**			
**Author**	**Process mapping methodology**	**Data collection: Who facilitated the pm ?**	**Data collection: Who participated in the unspecified pm methodology?**
Whitaker et al. (2024)	Not specified	Not specified	Local healthcare workers
Varghese et al. (2020)	Not specified	Not specified	Local healthcare workers
Gimbel et al. (2014)	Not specified	Researchers	Local healthcare workers
