# Supplementary material for: Process mapping in clinical healthcare settings in low-income and lower-middle-income countries: a scoping review
Source: Public Health Rev. 2026 Oct 14;47:1609105. doi: 10.3389/phrs.2026.1609105 (PMC13713439; doi:10.3389/phrs.2026.1609105)
Supplement: Supplementary file 4 [file DataSheet4.PDF]

Supplementary File 4: Data charting and coding

Coding tree

(For details on the coding process, see section “Data coding - Details” (P. 2)

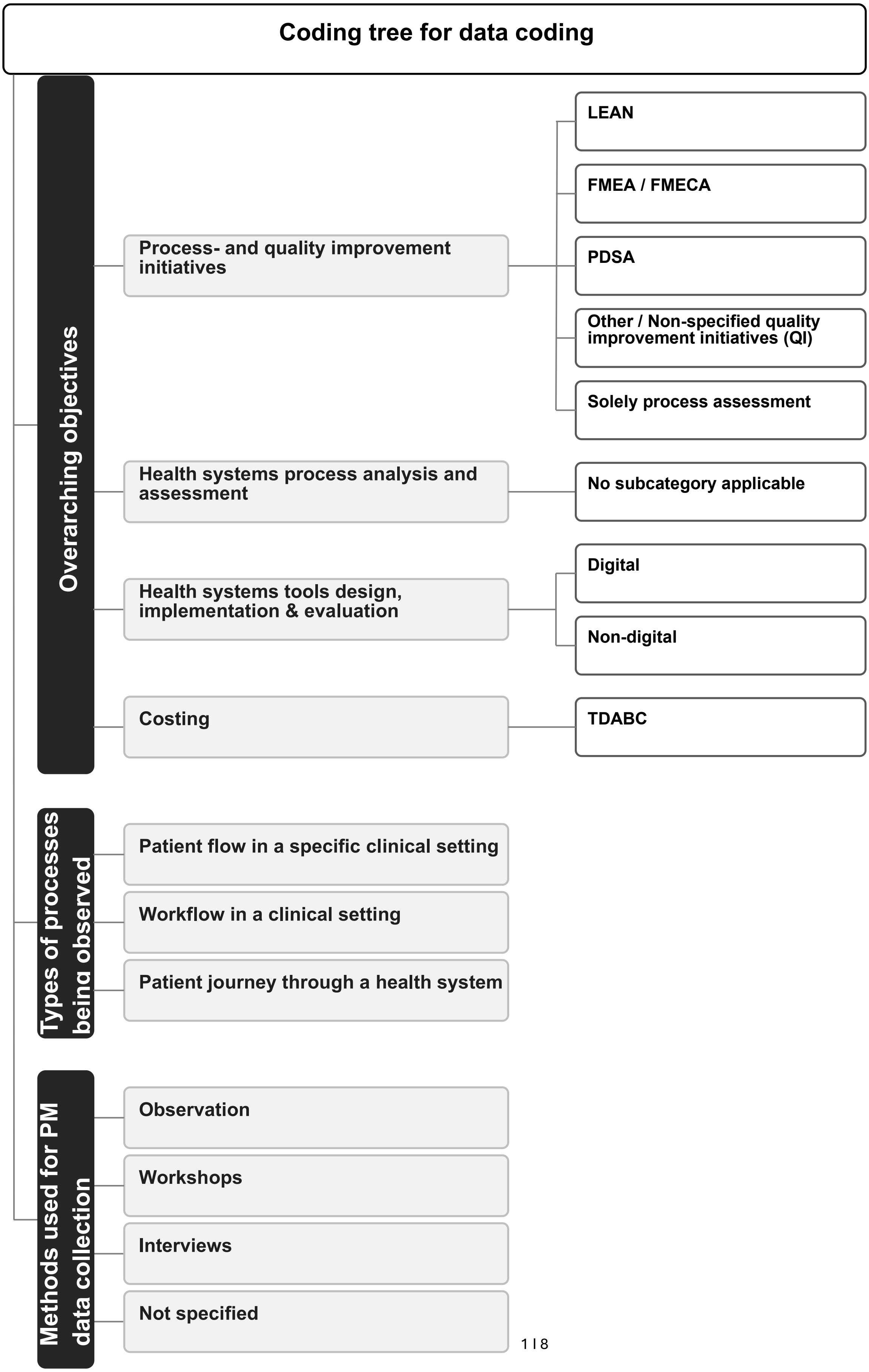

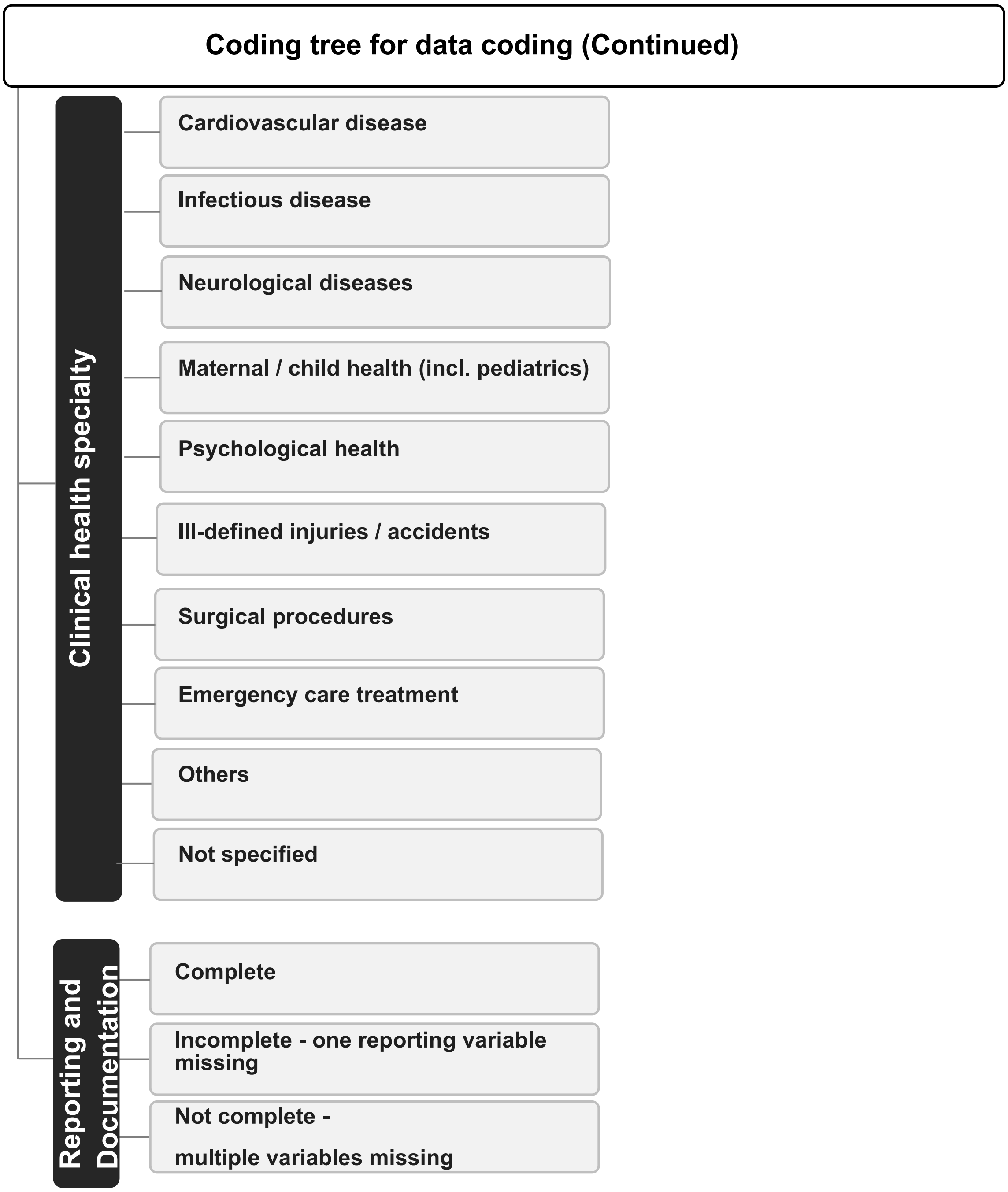

Data coding – Details

Coding - Overarching objectives
**Coding Details**; Studies and their textual data were analysed and narratively synthesised using a hybrid qualitative content analysis approach. Coding was based on predefined categories of the Framework by Antonacci et al. 2021, with additional categories developed inductively where required: “Health systems tools design / implementation / evaluation” adapted from Antonacci et al.; “Health systems process analysis and assessment” added
**Source/ Reference** Antonacci G, Lennox L, Barlow J, Evans L, Reed J. Process mapping in healthcare: a systematic review. BMC Health Serv Res. 2021 Apr 14;21(1):342. DOI: 10.1186/s12913-021-06254-1. PMID: 33853610; PMCID: PMC8048073. *(Table continued on the next page)*

Category	Decision rule for category	Subcategory	Codes
Process- and quality improvement initiatives	PM was primarily conducted as part of an explicit quality or process improvement initiative.<br>Primary aim to use PM to understand or improve healthcare processes using a structured QI methodology (PDSA, LEAN, FMEA) or to identify and implement improvement processes.	LEAN	Explicit Reference to Lean/Lean Six Sigma;<br>use of Lean tools incl. flow improvement, or efficiency increase
		FMEA / FMECA	Explicit reference to FMEA/FMECA; identification of failure modes, causes, and effects
		PDSA	Explicit reference to PDSA/PDCA cycles; use of testing of change ideas; use of measurement across cycles
		Others I Non-specified QI approach	Reference to other structured QI; Reference to process improvement without mentioning a specific method; Reference with aim of process improvement or quality improvement as purpose aim of the study or process mapping
		Solely Process Assessment	Mapping or description of sole process improvement or quality improvement assessment without reference to implemented change or explicit QI framework
Health systems process analysis and assessment	PM was used primarily as an analytical or descriptive tool to understand or assess processes at a macro or systems level (e.g. regional or cross-facility pathway).<br><br>Primary aim to use PM to generate insights into how a macro-level health system function- process assessment and or analysis and or evaluation of regional pathways, without an explicit link to a QI initiative, tool, development or cost estimation.	No Subcategory applicable	Reference of macro-level health system process assessment and/ or analysis and / or evaluation (regional pathways)
Health systems tools design, implementation and evaluation	PM was primarily used to support the design, implementation, or evaluation of a specific tool (Digital or non-digital) for a health system or service. PM was applied to map existing processes to inform tool design, development, implementation and / or evaluation.	Digital	Reference of design, implementation or evaluation of a digital solution / tool for a health system or service; e. g. adaptation of digital solutions (e.g., decision support, dashboards, eHealth platforms); process mapping used for digital tools development / evaluation
		Not digital	Reference of design, implementation or evaluation of a tool for a health system or service; process mapping used for analog tool development/ evaluation
Costing	PM was explicitly used within the Time-Driven Activity-Based Costing (TDABC) framework.	TDABC	Explicit reference and use of methodology ; Time-Driven Activity-Based Costing (TDABC); estimation of patient- or pathway-level costs linked to mapped processes

Coding - Various types of processes being observed

Coding Details: All included studies and their textual data in regards to their process mapping activities were extracted and read repeatedly. An inductive qualitative content analysis was performed through: open coding, grouping similar descriptions of mapped processes. Through discussion within the study team, preliminary codes were grouped into these three descriptive categories. For each category a decision rule was formed, analysed and synthesized narratively and coded into the three categories:

Main coding question: What type of process did the authors map?

Category	Decision rule for category	Category Interpretation	Code
Patient flow in a specific clinical setting	Mapping of a patient movement within one clinic setting described	- Focus on patient movement within a single clinical setting incl. entry, exit points and sequential care steps<br>- Emphasis on intra-facility / organisation flow and patient treatment process through care	- Patient flow from arrival to discharge<br>- Flow of patient within the facility<br>- Care cycle<br>- Sequential care steps<br>- Patient flow / treatment process from entering clinical setting to exiting the setting<br>- Patient movement and management between departments at the hospital
Workflow in a clinical setting	Mapping of workflows described from a work perspective, focus on work process within a clinical setting	-Focus on work processes of healthcare teams / workers within a setting<br>-Emphasis on professional activities / task sequences<br>-Patient movement mentioned but process map centres on professional activities and organisational processes mainly<br>- Steps of a process from worker / provider perspective	- Work process of main steps<br>- Work process of treatment with a sickness / illness<br>- Admission / Discharge process<br>- Clinical work procedures / SOP / Team workflow
Patient journey through a health system	Mapping of patient pathways across multiple facilities or level of care described	- Focus on macro level patient journey across multiple service or levels of care often to a specific condition or treatment<br>- Navigation between facilities<br>- System- level coordination<br>- Emphasis on transitions and continuity of care across a health system	- Referral pathways<br>- Cross- facility movement,<br>- Navigation between hospitals, system-level coordination<br>- Multi- centre care<br>- Process mapping focus on transitions.

Coding - Methods used for PM data collection

Coding Details: In studies where two or more methods were used for data collection: the different methods were analysed separately from each other.

Category	Description	Decision rule for category
Observation	Direct observation of processes or activities	*Paper mentions collecting data for their process map with observations*
Workshops	Workshops, meetings, stakeholder sessions)	*Paper mentions collecting data for their process map with workshops/meetings*
Interviews	Individual or group interviews with participants (e.g., staff, patients)	*Paper mentions collecting data for their process map with interviews*
Not specified	PM data collection conducted but methodology not clearly described.	*Coded as “non specified” if not clearly reported*

Coding - Clinical health speciality

Details: Categories were primarily derived from existing classification frameworks (WHO, Davies et al.), and additional categories were developed inductively for three categories as they could not be categorised to the existing frameworks.

Source/ Reference: 1) Ralaidovy AH, Adam T. Standard classification of health categories. Global Observatory on Health R&D, World Health Organization, 2022 2) Davies EL, et al. Reporting and conducting patient journey mapping research in healthcare: A scoping review. J Adv Nurs. 2023 Jan;79(1):83-100. doi: Davies EL, Bulto LN, Walsh A, Pollock D, Langton VM, Laing RE, Graham A, Arnold-Chamney M,

Category	Description	Decision rule for category	Source
Cardiovascular disease	Heart and vascular conditions	Paper mentions cardiovascular disease as their main investigation / focus	*WHO / Ralaidovy AH and Adam T. (2022)1*
Infectious disease	Communicable diseases (e.g., HIV, TB)	Paper mentions infectious disease as their main investigation/ focus	*WHO / Ralaidovy AH and Adam T. (2022)1*
Neurological diseases	Disorders of the nervous system	Paper mentions neurological disease/ treatment as their main investigation/ focus	*WHO / Ralaidovy AH and Adam T. (2022)1 / adapted*
Maternal / Child health (incl. Pediatrics)	Maternal, neonatal, or pediatric care	Paper mentions mother/ child health, maternal health or pediatric health as their main investigation/ focus	*WHO / Ralaidovy AH and Adam T. (2022)1 / adapted*
Psychological health	Mental and behavioral health	Paper mentions psychological distress/ issues/ diseases as their main investigation/ focus	*Davies et al (2023)2*
Ill defined injuries / accidents	Trauma or injury-related care	Paper mentions ill-defined injuries/ accidents as their main investigation/ focus	*WHO / Ralaidovy AH and Adam T. (2022)1*
Surgical procedures	Operative and perioperative care	Paper mentions surgical procedures as their main investigation/ focus	*Davies et al (2023)2*
Emergency Care Treatment	Emergency care without specific diagnosis	Paper mentions emergency care treatment as their main investigation/ focus without specifying one particular disease/ treatment	*added / adapted*
Others	Not fitting predefined categories	Paper mentions other treatment/ disease as their main focus	*added / adapted*
Not specified	No clear clinical focus reported	Paper does not mention a specific clinical health speciality as their focus / a category cannot be specified	*added / adapted*

Coding - Reporting and Documentation

Details:
For reporting and documentation, we assessed whether all of our **predefined reporting variables** were described in each study. Data were initially coded as complete or not complete; during charting we refined this scheme to distinguish studies with only one missing reporting item from those with more gaps in reporting.

Category methodology:	Description	Decision rule for Category
Complete	Reporting and documentation of all variables and pm activities surrounding the process mapping was documented and clearly described	*All predefined variables to extract for the extraction sheet are reported and documented.*
Incomplete- Only one reporting variable missing	Exactly one reporting item missing or not specified; missing item is reported in extraction sheet	*Category introduced during charting as only one item missing occurred often.*
Not complete- multiple variables missing	Two or more items / variables are missing or not specified.	*Assigned when more than one predefined variable to extract for the extraction sheet was not documented / not clearly described or reported.*

Data charting

Variable	Operational definition	Response options	Assumption/ Specification
Authors (year of publication)	Name first author et al. (Year paper publication)	-	-
Title Article	Full title of included study	-	-
Research Method	Overall methodological approach,	Multi-Method<br>Qualitative<br>Quantitative	-
Name Journal	Journal in which study was published	-	-
Country study conducted	Country or countries in which the study was conducted	-	Multiple responses were allowed where applicable
Health system level	Level of care involved	Tertiary healthcare<br>Secondary healthcare<br>Primary healthcare<br>Other<br>Not specified	Multiple responses were allowed where applicable
Years of data collection	Time period in which data were collected	-	-
Health care settings	Settings in which the mapped process occurred	Outpatient; Inpatient; OT; Emergency Room; Other (Homecare); Not Specified; Multiple Settings	Multiple responses were allowed where applicable<br>Coded as "non specified" if not clearly described.<br>Multi-Settings (>3) were collapsed; when no specification was possible<br>Other options if n=1
Clinical health speciality	Main clinical area / speciality addressed by the study	Cardiovascular disease;<br>Infectious disease;<br>Neurological diseases;<br>Maternal / Child health (incl. Pediatric; Psychological health; Ill defined injuries / accidents ;<br>Surgical procedures;<br>Emergency Care Treatment;<br>Others (home care);<br>Not specified	Multiple responses were allowed where applicable; Others if n=1<br>See coding framework
Overarching objectives	Primary purpose of the process mapping exercise / activity	Process- and quality improvement initiatives;<br>Health systems process analysis and assessment;<br>Health system tools design, implementation, evaluation;<br>Costing	See coding
Type of processes being observed	What type of processes was process mapped?	Workflow in a clinical setting; Patient journey through a health system; Patient flow in a specific clinical setting	See coding
Methods used for PM data collection	Method used to construct the process map	Observation; Interviews; Workshops; Observation & Workshops; Observation & Interviews;<br>Not specified	See coding<br><br>Combined categories were used where multiple methods contributed to the map generation<br><br>Coded as "non specified" if not clearly described.
Preparation	Whether the study reported preparatory activity before starting process mapping	No; Yes; Not specified	Coded as "non specified" if not clearly described.
Data collection approach	Description of how process mapping data were collected and by whom	-	See specific subcategory
Local HCW Involvement	Whether local healthcare workers were involved in the mapping process	Yes;<br>No;<br>Not specified	Coded as yes / no only if explicitly stated.<br><br>Coded as "non specified" if not clearly described.
Patient representative	Whether patients or patient representatives were involved	Yes;<br>No;<br>Not specified	Coded as yes / no only if explicitly stated.<br><br>Coded as "non specified" if not clearly described.

Variable	Operational definition	Response options	Assumptions / Specification
Process map generation	Whether and how the map was generated with participants or stakeholders	Yes, during;<br>Yes after;<br>Yes, but unclear how;<br>No; Not specified	Unclear specification where labelled as yes, but unclear how.<br><br>Coded as “non specified” if not clearly described.
Process map analysis	Whether and how the process map was analysed ?	Yes, during;<br>Yes after;<br>Yes, but unclear how;<br>No;<br>Not specified	Coded as present only if explicitly stated.<br><br>Coded as “non specified” if not clearly described.
Validation final pm with local	Whether the final process map was validated with local healthcare workers or stakeholders	Yes;<br>Not specified<br>Not specified but pm generated with local healthcare workers	Coded as present only if explicitly stated.<br><br>Coded as “non specified” if not clearly described.
Take it forward	Whether findings informed implementation, improvement work or further system improvements have been applied	Yes, implemented;<br>No, but identified process or system challenges for improvement;<br>Not specified	Coded as; yes when explicit follow- up use and implementation were reported.<br><br>Coded as Not but identified.. if stated that process or system challenges were identified.<br><br>Coded as “non specified” if not clearly described.
Reporting and Documentation	To what extent were all predefined extraction variables reported in the article, and were any of these variables missing?	Complete<br>Only one reporting variable missing –(incl. details)<br>Not complete- multiple variables missing	See coding

PM Data collection - Workshop

Variable	Operational definition	Response options	Assumptions / Specification
**PM data collection Workshop**	Workshop or Meetings as data collection to inform PM was conducted	Workshops	See Coding
**Workshop:**<br>Who facilitated in the pm workshop	Identifies who facilitated or led the process-mapping workshop.	Researchers; Not specified;<br>Local healthcare workers	“Researchers” was used when workshop facilitation was led by the research team or article authors.<br>“Local healthcare workers” was used when facilitation was led by local healthcare workers<br>“Not specified” was used when the publication did not clearly identify the facilitator.
**Workshop:**<br>Who participated in the pm workshop?	Identifies who participated in the workshop.	Local healthcare workers;<br>Researchers<br>Not specified	Local healthcare workers was used when local healthcare workers were participating in the workshop; Researcher when researcher were participating in the workshop, additionally to the leading part.

PM Data collection - Observation

Variable	Operational definition	Response options	Assumptions / Specification
**PM data collection: Observation**	Observation as data collection to inform PM was conducted	Observations	See Coding
**Observation:**<br>Who collected the data during observation	Identifies who collected the data.	Researchers;<br>Local healthcare workers;<br>Local healthcare workers as researchers/ authors;<br>Not specified	“Researchers” was used when observation was conducted by the research team or article authors.<br><br>“Local healthcare workers” was used when observation led by local healthcare workers<br><br>“Local healthcare workers as researchers/authors” was used when local healthcare workers were explicitly described as undertaking observation in a formal researcher or author role.<br><br>“Not specified” was used when the publication did not clearly identify who conducted the observation.

Variable	Operational definition	Response options	Assumptions / Specification
**Observation:** Additional contribution during observation?	Identifies if and who additionally contributed to the data observation process	Local healthcare workers;<br>Researchers;<br>Not specified	"Researchers" was used when additional contribution was a researcher or article author member.<br><br>Local healthcare worker was used when additional contribution was a local healthcare worker<br><br>"Not specified" was used when the publication did not clearly specify. .

PM Data collection - Interviews

Variable	Operational definition	Response options	Assumptions / Specification
**PM data collection: Interviews**	Interviews as data collection method to inform PM was conducted	Interviews	See Coding
**Interviews:** Who conducted the interviews?	Identifies who, with which roles conducted the interviews	Researchers;<br>Local healthcare workers;<br>Not specified	"Researchers" was used when interview was conducted by the research team or article authors.<br><br>"Local healthcare workers" was used when interview was led by local healthcare workers<br><br>"Not specified" was used when the publication did not clearly identify the facilitator.
**Interviews: Who** were interviewed?	Describes who with which role were interviewed	Local healthcare workers<br>Researcher<br>Not specified	"Researchers" was used when the interviewee was a researcher or article author member.<br><br>Local healthcare worker was used when the interviewee was a local healthcare worker<br><br>"Not specified" was used when the publication did not clearly specify. .

PM Data collection - Not specified

Variable	Operational definition	Response options	Assumptions / Specification
**PM data collection: Not specified**	Indicates that process-mapping data were collected but the methodology was not clearly specified	Not specified	See coding
**Not specified:** Who facilitated the pm?	Identifies reported facilitators of process-mapping activities	Not specified; Researchers<br>Local healthcare workers	"Researchers" was used when pm was conducted by the research team or article authors.<br><br>Local healthcare worker was used when pm was conducted by the research team or article authors.<br><br>"Not specified" was used when the publication did not clearly identify the facilitator.
**Not specified:** Who participated in the unspecified pm methodology?	Describes participants involved in process mapping activities	Not specified;<br>Researcher;<br>Local healthcare workers	"Researchers" was used when pm was conducted by the research team or article authors.<br><br>Local healthcare worker was used when pm was conducted by the local healthcare worker.<br><br>"Not specified" was used when the publication did not clearly identify the facilitator.
